# Deubiquitinases in Cancers: Aspects of Proliferation, Metastasis, and Apoptosis

**DOI:** 10.3390/cancers14143547

**Published:** 2022-07-21

**Authors:** Jiaqi LIU, Chi Tim LEUNG, Luyun LIANG, Yuqin WANG, Jian CHEN, Keng Po LAI, William Ka Fai TSE

**Affiliations:** 1 Key Laboratory of Environmental Pollution and Integrative Omics, Education Department of Guangxi Zhuang Autonomous Region, Guilin Medical University, Guilin 541004, China; liujiaqi@glmu.edu.cn (J.L.); rony_lirong@glmc.edu.cn (L.L.); 122006017@glmc.edu.cn (Y.W.); kengplai@cityu.edu.hk (K.P.L.); 2 Department of Chemistry, City University of Hong Kong, Hong Kong SAR, China; timleung@cityu.edu.hk; 3 Guangxi Key Laboratory of Tumor Immunology and Microenvironmental Regulation, Guilin Medical University, Guilin 541004, China; 4 Laboratory of Developmental Disorders and Toxicology, Center for Promotion of International Education and Research, Faculty of Agriculture, Kyushu University, Fukuoka 819-0395, Japan

**Keywords:** proliferation, metastasis, apoptosis

## Abstract

**Simple Summary:**

This review summarizes the current DUBs findings that correlate with the most common cancers in the world (liver, breast, prostate, colorectal, pancreatic, and lung cancers). The DUBs were further classified by their biological functions in terms of proliferation, metastasis, and apoptosis. The work provides an updated of the current findings, and could be used as a quick guide for researchers to identify target DUBs in cancers.

**Abstract:**

Deubiquitinases (DUBs) deconjugate ubiquitin (UBQ) from ubiquitylated substrates to regulate its activity and stability. They are involved in several cellular functions. In addition to the general biological regulation of normal cells, studies have demonstrated their critical roles in various cancers. In this review, we evaluated and grouped the biological roles of DUBs, including proliferation, metastasis, and apoptosis, in the most common cancers in the world (liver, breast, prostate, colorectal, pancreatic, and lung cancers). The current findings in these cancers are summarized, and the relevant mechanisms and relationship between DUBs and cancers are discussed. In addition to highlighting the importance of DUBs in cancer biology, this study also provides updated information on the roles of DUBs in different types of cancers.

## 1. Introduction

### 1.1. Ubiquitination

Ubiquitination is a reversible post-translational modification process that involves small protein, ubiquitin (UBQ) [1]. The process can be divided into three stages. UBQ is first activated by E1 ubiquitin-activating enzyme in an ATP-dependent manner. Then, the activated UBQ is transferred to the E2 ubiquitin-conjugating enzyme. Subsequently, the process completes with the E3 ubiquitin-protein ligase, specifically bound to a substrate protein, that recruits the ubiquitin-E2 complex. Such interactions lead to the transfer and conjugation of UBQ to the lysine residue in the target substrate via isopeptide bonds [1,2]. The UBQ chain is formed by the addition of UBQ to the lysine residue of the previous UBQ on the UBQ-conjugated substrate. This polyubiquitylated complex is subjected to proteasomal degradation, lysosomal degradation, or autophagocytosis. It is also known to be involved in other cellular functions such as modifying kinase activities and activating transcriptional factors [3,4].

### 1.2. Deubiquitination

Deubiquitinases (DUBs) deconjugate UBQ from ubiquitylated substrates to regulate its activity and stability [5]. Approximately 100 DUBs have been identified in humans and can further be divided into two groups according to their catalytic activities: (a) cysteine protease DUBs and (b) metalloprotease DUBs [6,7]. Cysteine protease DUBs consist of seven subclasses based on their distinct enzymatic domains: ubiquitin-specific proteases (USPs), ovarian tumor proteases (OTUs), ubiquitin carboxyl-terminal hydrolases (UCHs), Machado-Joseph disease proteases (MJDs), motif interacting with ubiquitin-containing novel DUB family (MINDY), zinc-finger and USFP domain protein (ZUFSP), and monocyte-chemotactic protein-induced protein (MCPIP) family [8,9,10]. DUBs undergo deubiquitination via nucleophilic attack by their catalytic cysteine on the isopeptide bond [11]. Finally, the zinc-dependent JAB1/MPN/MOV34 metalloprotease (JAMMs) family belongs to the metalloprotease DUBs family, in which the two zinc ions at the catalytic site of the DUB could activate a water molecule, and thus form a hydroxide ion to attack the isopeptide bond [12]. The major roles of DUBs can be categorized into three categories. First, they help generate and recycle free UBQ. DUBs process inactive ubiquitin precursors that are either fused with ribosomal proteins or translated as ubiquitin polymers into active and free ubiquitin monomers. In addition, before subjecting the ubiquitylated protein to degradation, DUBs play an important role in deconjugating UBQ from the substrate to prevent degradation of UBQ and maintain UBQ homeostasis. Second, DUBs can modify ubiquitination by switching between degradative and non-degradative signals by editing the ubiquitin chains. Third, DUBs play an important role in rescuing proteins and maintaining their stability. They antagonize the actions of E3 ligase by cleaving the isopeptide linkage, thus deconjugating UBQ from the ubiquitylated protein. Deubiquitination can also act as a proof-reading mechanism to rescue inappropriately ubiquitylated proteins during degradation. Moreover, DUBs regulate the stability of proteins, such as E3 ligase, which is targeted for self-ubiquitination [5,13,14].

### 1.3. Biological Functions of DUBs and Their Expressions in Selected Organs

DUBs exert crucial functions in various cellular processes such as cell cycle regulation, DNA damage response, cell proliferation, and apoptosis. Ectopic expression of DUBs leads to pro- or anti-tumorigenic effects during cancer progression. Furthermore, DUBs can act as cancer biomarkers or therapeutic targets to aid in clinical prognosis and treatments [14,15,16]. Due to its importance in various biological functions, the number of DUB works that has been deposited in the PubMed accessed on 20 July 2022 (https://pubmed.ncbi.nlm.nih.gov/) has been doubled in these ten years (505 publications in 2012 to 1039 publications in 2021 by searching the word “Deubiquitinases”). Although numerous reviews have focused on the relationship between DUBs and cancer, there is limited organized information on the roles of DUBs grouped by type of cancer. It would be informative to summarize the roles of DUBs in different types of cancers that are prevalent worldwide. 

Proliferation, metastasis, and apoptosis are the three major hallmarks of cancers [17,18]. Deregulated cell cycle could lead to changes in cell proliferation. The cell cycle can be divided into the G1 (cell growth), S (DNA synthesis), G2, and M (cell division) phases. It is tightly regulated by various checkpoints controlled by cyclins and cyclin-dependent kinases (CDKs) [19]. E3 ligases are known to participate in almost every phase; thus, their regulation through DUBs is expected [20,21]. For example, USP17 controls the G1 phase; USP10, USP14, USP17, and BAP1 play roles in the G1/S transition; and USP7 is involved in the mitotic phase [16]. In addition, cancer cells have the ability to evade apoptosis [22], and DUBs can target different pro- and anti-apoptotic proteins in both extrinsic and intrinsic pathways [23,24]. DUBs are known for their regulatory roles in different cell signaling pathways, such as androgen receptor (AR), estrogen receptor (ER), Wnt/β-catenin, and p53 signaling, which are related to proliferation and apoptosis [25,26]. The tumor suppressor p53 is a transcription factor that prevents genomic mutations and plays a protective role in tumor onset and progression. It can be regulated by ubiquitination, indicating the importance of DUBs in controlling the ubiquitin cycle [27]. Different DUBs target both MDM2 and p53. The p53 pathway is highly related to MDM2, and its stability can be regulated by DUBs, such as USP2 and USP7 [28,29]. In addition, DUBs directly target p53 or p53-associated proteins, leading to cell proliferation. For example, USP10 and USP29 can directly deubiquitinate p53 [30,31], while USP5 regulates p53 levels and alters cell growth that is associated with p21 induction [32]. Another feature of cancer is metastasis, which is the ability of cancer cells to spread to different tissues or organs. Numerous DUBs regulate epithelial-mesenchymal transition (EMT) transcription factors [16,23]. Metastasis is a series of biological processes that includes numerous invasion-metastasis cascades. EMT refers to a change in different adhesion molecules in cells, thus adopting migratory and invasive behavior [33]. It is a critical invasive process in cancer metastasis [34]. DUBs, such as OTUB1 and USP37, target SNAIL [35,36], while USP9X targets SMAD4 [37]. In addition, other DUBs, such as PSMD14, could target other molecules, such as growth factor receptor-bound protein 2 (GRB2) [38]. The current review, instead of describing the detailed mechanisms of each DUBs, looks at the issue from another angle. We present a general summary of the roles of selected DUBs in the cell cycle, proliferation, apoptosis, and metastasis in specific types of cancer. 

**Figure 1:**
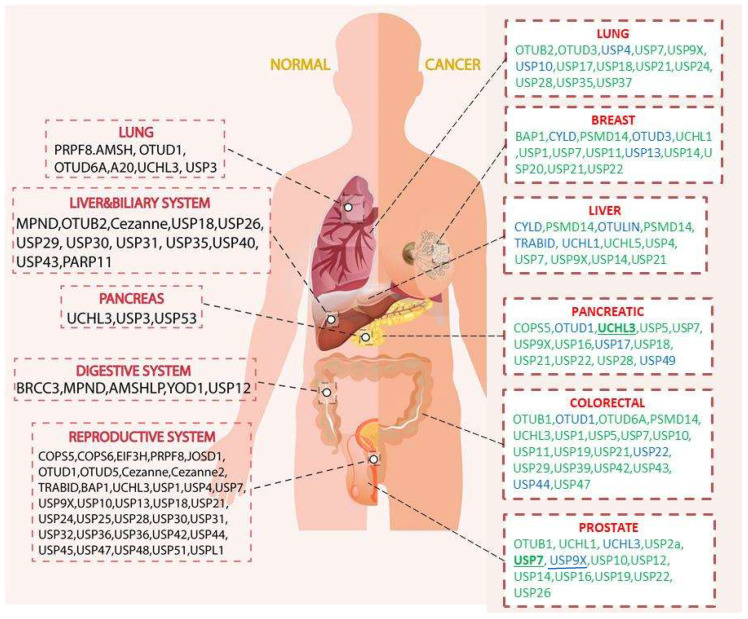
Deubiquitinases (DUBs) in different organs. The figure is divided into two panels, the normal condition (left), and the cancer (right). The normal condition is based on the [7], and the inclusion criteria are limited to the DUBs that are significantly overexpressed, relative to their mean expression in other tissues. Five systems/organs are selected, which are the lung, liver and biliary system, pancreas, digestive system, and reproductive system. While the cancer (lung, breast, liver, pancreatic, colorectal, prostate) related DUBs are summarized from the literatures in this review. Green indicates the DUBs that are highly expressed in the cancers tissue; while blue indicates the supressed expression. It should be noted that such expression levels are various in studies, the over or supressed DUBs expression are presented as a general trend from the works in the literature examined in this review. Comparing the two conditions, it is limited to spot the common DUBs (underlined) in both conditions. Such observation implies that the highly expressed DUB in particular tissue is not necessary to be the major DUB that contributes to cancer progression.

First, we displaced a figure that extracted the information from a review paper that summarized the highly expressed DUBs in organs in the left panel [7], while the right panel summarizes the deregulated DUBs in cancers that we described in this review (Figure 1). Through this comparison, it should be noted that the high expression level of DUBs in normal tissue was not necessarily linked to the related cancers. We discuss cancer-related DUBs in the following sections.

## 2. Liver Cancer

Hepatocellular carcinoma (HCC) is the third leading cause of cancer-related mortality worldwide [39]. HCC is related to underlying chronic liver disease and other factors such as excessive alcohol intake, hepatitis virus infection, obesity, and nonalcoholic fatty liver disease (NAFLD) [40]. The modulation of immune and inflammatory responses is closely linked to cancer [41].

### 2.1. Inflammation

Inflammation is recognized as one of the hallmarks of cancer owing to its promoting role in neoplastic transformation [18,42,43,44]. Chronic inflammation caused by hepatitis virus infection or hepatocyte injury is a major cause of liver cancer [45]. DUBs that regulate inflammatory responses have been suggested to play a role in cancer development.

CYLD is a well-known DUBs that is involved in cancer formation and is downregulated in both tumor tissues and HCC cell lines compared to primary non-cancerous hepatocytes [46,47]. Using a mouse model with deletion of liver-specific CYLD exon7/8 (*CYLD^FF^xAlbCre*), Urbanik et al. demonstrated increased biliary injury, liver fibrosis, and susceptibility to diethylnitrosamine phenobarbital (DEN/PB)-induced liver tumor development. The anti-inflammatory role of CYLD is further supported by an increase in T cell and macrophage infiltration and elevated mRNA expression of inflammation-related genes in the liver via nuclear factor κB (NF-κB) signaling [48]. Previous studies indicated that CYLD could act as a negative regulator of NF-κB signaling by deubiquitinating NF-κB essential modulator (NEMO), the subunit of IκB kinase (IKK), and tumor necrosis factor receptor-associated factor (TRAF) 2 and 6 [49,50]. Furthermore, CYLD deubiquitylated mitogen-activated protein kinase kinase kinase 7 (TAK1), which negatively regulates TAK1-JNK-p38 signaling. It also mitigated inflammation and fibrosis in non-alcoholic steatohepatitis (NASH)-induced mice and monkeys [51]. Another DUB, OTULIN, also suppresses inflammation. This is the only DUB that specifically removes the linear UBQ chain. Ablation of OTULIN in mice induces liver inflammation and fibrosis, leading to neoplastic lesions and liver cancer [52]. Similarly, mice with hepatocyte-specific OTULIN deletion (*Otulin*∆hep) showed severe liver complications such as NF-κB-independent inflammation, growth of dysplastic nodules, NASH- and cirrhosis-like diseases, and HCC [53].

### 2.2. Cell Proliferation

Numerous DUBs are known to be involved in cell proliferation. The expression of PSMD14, USP14, and USP21 was upregulated in HCC patients and correlated with a shorter overall survival rate [38,54]. PSMD14 was reported to promote cell proliferation in vitro and larger xenograft tumor formation in vivo [38]. USP21 deubiquitinated and stabilized MEK2, thus activating the ERK pathway for cell proliferation, colony formation, and cell cycle progression, and promoted tumor growth [54]. USP14 activates phosphatidylinositol-3 kinase (PI3K) via the Wnt/β-catenin-mediated pathway [55,56].

In contrast, several DUBs were downregulated in HCC, suggesting their anti-proliferative roles. UCHL1 has been reported to be downregulated in HCC cell lines compared with normal tissues. Suppression of colony formation and cell proliferation with increased G2/M cell cycle arrest was observed in cells transfected with UCHL1-expressing constructs [57]. Moreover, CYLD^-/-^ mice show increased sensitivity to diethylnitrosamine-induced hepatocarcinogenesis via increased c-Jun N-terminal kinase 1 (JNK1)-mediated cell proliferation. CYLD knockout increases ubiquitination of TRAF2, leading to sustained JNK signaling, which subsequently upregulates cyclin D1 and c-Myc expression [46]. USP7 deubiquitinate p53 and modulate the p53-Mdm2 pathway [58,59,60]. It can be recruited by the scaffold protein Abraxas brother 1 (ABRO1), leading to p53 deubiquitination and stabilization, thus suppressing in vitro clone formation in HCC HepG2 [61]. Furthermore, miRNA regulation of DUB has been suggested. It has been shown that miR205 could suppress USP7, resulting in increased cell proliferation via downregulation of p53 and its downstream targets, such as p21, p27, and growth arrest and DNA damage-inducible protein (GADD45) [29].

### 2.3. Migration, Invasion & Metastasis

Various DUBs are known for their metastatic roles in HCCs. For example, UCHL5 is highly expressed in liver cancer tissues. Cell line studies have revealed that it could promote cell migration and invasion by deubiquitinating pre-mRNA processing factor 19 (PRP19) [62]. Moreover, USP4 was reported to promote migration, invasion, and EMT by deubiquitinating phosphoinositide 3-kinases (PI3K) and activate transforming growth factor beta (TGF-β) pathways by deubiquitinating TGF-β receptor 1 (TGFR1) [55,63]. Another DUB, USP9X, deubiquitinated and stabilized survivin protein, and contributed to cell proliferation and invasion in HCC cells via the long noncoding RNA LNC473 [64]. Moreover, suppression of USP9X by miR-26b leads to SMAD4 downregulation and attenuation of cell migration and EMT [37]. Furthermore, DUB PSMD14 enhances cell migration and invasion in vitro via deubiquitinating GRB2, which is related to tumor metastasis [65]. PSMD14 expression also positively correlated with vascular invasion in HCC patients [38]. Moreover, injection of PSMD14-expressing HCC cells resulted in increased lung metastases in the nude mouse xenograft model. PSMD14 has a positive role in migration and invasion by deubiquitinating TGF-β receptors and caveolin 1 (CAV1), resulting in the activation of TGF-β signaling [66]. In contrast, TRABID (ZRANB1) expression is downregulated in HCC tumor tissues. Reintroduction can reduce HCC progression and metastasis. Furthermore, overexpression of TRABID reduced EMT markers in HCC cells through Akt-mediated TRABID phosphorylation, which further deubiquitinated Twist and promoted its degradation [67].

### 2.4. Apoptosis 

**Figure 2:**
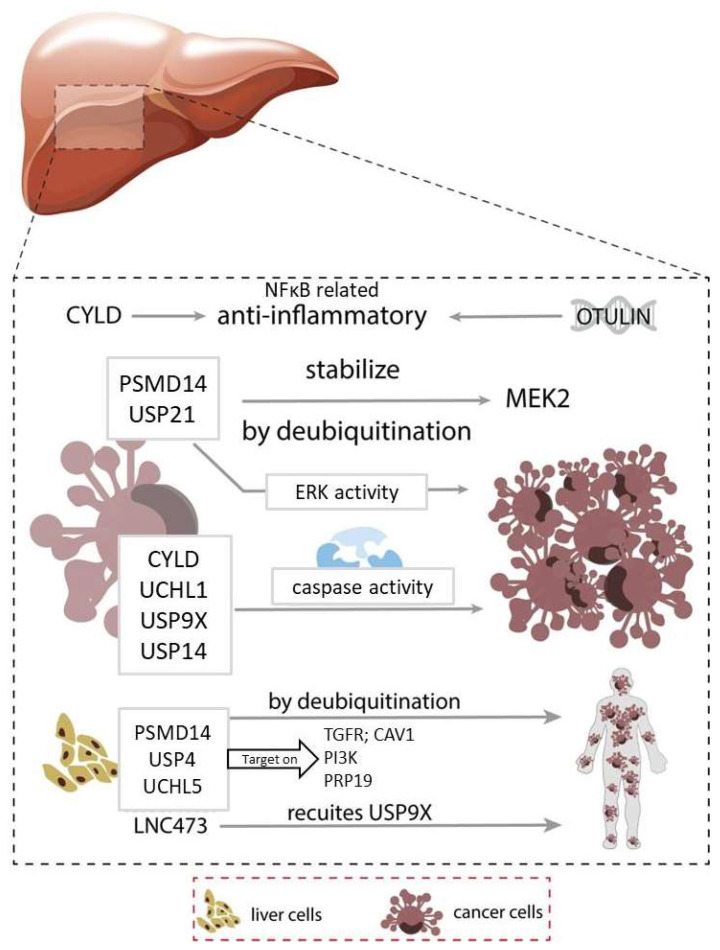
Roles of deubiquitinases (DUBs) in liver cancer. Modulations of the immune and inflammatory responses are closely related to the liver cancer. CYLD and OTULIN act as the anti-inflammatory factors that are linked to the NFκB pathway. Other DUBs such as PSMD14, and USP21 could stabilize the MEK2 and thus regulate the cell proliferation. On the other hand, DUBs such as CYLD, UCHL1, USP9X, and USP14 could regulate the caspase activities. Moreover, PSMD14, USP4, and UCHL5 could target on TGFR, PI3K, and PRP19 respectively and influence the metastasis. Detailed mechanisms were described in the main text.

CYLD siRNA in a HCC cell line suppressed apoptosis via the NFκB-mediated tumor necrosis factor α signaling pathway by lowering caspase-3 activity [68]. Similarly, UCHL1 influences caspase-dependent pathways and promotes apoptosis. The tumor suppressor p53 can be further deubiquitinated and stabilized by UCHL1 and USP9X [57,69]. USP9X also promoted apoptosis in HCC by deubiquitinating apoptotic signaling-regulating kinase (ASK1) and enhancing oxidative stress-induced JNK activation and cell death [70]. GSK-3β suppressed reactive oxygen species (ROS)-induced apoptosis by suppressing ASK1 protein expression. Upon treatment with a GSK-3β inhibitor, ASK1 levels were not recovered if the USP9X gene was silenced, implying the critical role of USP9X in stabilizing ASK1 in HCC [71]. In contrast, USP14 promoted tumor progression and suppressed apoptosis in HCC via the downregulation of caspase-3 and upregulation of BCL-2 protein [56]. A summary is presented in Figure 2.

## 3. Breast Cancer

Breast cancer is the leading malignant tumor in women worldwide [72]. Patients with breast cancer experience undesirable metastasis, relapse rates, and drug resistance [73]. The World Health Organization (WHO) data mentioned that it had 2.26 million new cases in 2020, which ranked it as the most common cancer.

### 3.1. ERα Signaling

Breast cancers are highly related to the estrogen receptor ERα (ER^+^), which accounts for approximately 70% of cases [74]. Binding of estradiol (E2) to ERα activates downstream pathways and causes tumorigenesis [75]. Several DUBs, such as USP1, USP7, and USP22, have been shown to play regulatory roles in ERα signaling. They deubiquitinate ERα and activate its relative signaling pathway to promote ERα^+^ breast cancer development [76,77,78]. USP11 positively regulates ERα transcriptional activity in breast cancer cells in an E2-dependent manner [79]. On the other hand, high expression of UCHL1 was found to be inversely correlated with survival rate, in which UCHL1 deubiquitinated the epidermal growth factor receptor, thus suppressing ERα gene transcription, leading to resistance to anti-estrogen therapy in treating breast cancer [80].

### 3.2. Cell Proliferation

DUBs have been reported to promote cell proliferation and cell cycle progression in breast cancer cells. BAP1 is well-known for its role in breast cancer development. It deubiquitinates Krüppel-like factor 5 (KLF5), which is highly expressed in ERα-negative basal subtype breast cancers [81,82,83]. In KLF5-positive breast cancer cells, BAP1 reduced p27 expression and promoted cell proliferation in vitro. It also promoted in vivo xenograft tumor growth by stabilizing KLF5 [84]. Despite being anti-proliferative in ER^+^ breast cancer by inhibiting ERα signaling [85], the AR promotes cell proliferation in ER-breast cancers [86,87,88]. USP14 is deubiquitinated and impedes AR degradation. Inhibition of USP14 resulted in suppressed AR-responsive (AR+) breast cancer cell proliferation by G0/G1 cell cycle arrest [89]. Similarly, other DUBs, such as PSMD14 and USP21, alter cell cycle regulation. PSMD14 knockdown resulted in G0/G1 arrest, reduced expression of cyclin D1, and attenuated cell proliferation [90], whereas USP21 deubiquitinated and stabilized the transcription factor forkhead box M1 (FOXM1), which is crucial for G2/M transition [91]. The depletion of USP21 in breast cancer cell lines resulted in cell cycle delay and mitigated cell proliferation [92]. It was further reported to regulate the cell cycle via deubiquitinated FOXM1, which is suggested to be one of the master regulators in cancers [93]. Moreover, the deubiquitination of cell cycle-associated cyclin D1 by USP11 has been suggested to be associated with poor survival in ERα^+^ breast cancer patients [79]. 

### 3.3. Migration, Invasion & Metastasis

UCHL1 is highly expressed in aggressive breast cancer. It induced cell migration in an in vitro model and extravasation in in vivo zebrafish and mouse xenograft models. UCHL1 promoted TGFβ-SMAD signaling by deubiquitinating the TGFβ type I receptor and SMAD2, resulting in enhanced metastasis [94]. Another DUB, USP20, promoted transwell migration and invasion through the deubiquitination of SNAIL family transcriptional repressor 2 (SNAI2). Intravenous injection of USP20 siRNA breast cancer cells into mice resulted in reduced lung colonization and nodules, and this effect could be rescued by SNAI2 overexpression [95]^.^ Moreover, PSMD14 was upregulated in breast cancer tissues and found to be associated with clinical tumor stage and poorer overall survival. It plays a role in pro-tumorigenesis in cancers, and its relative knockdown experiment in breast cancer cell lines further confirmed its role in cell migration and the EMT process [96,97,98,99]. In contrast, some DUBs have been labeled as anti-metastatic in breast cancers. For example, CYLD expression was downregulated in breast cancer. Its overexpression reduced cell viability and migration via NF-κB and receptor activator of nuclear factor kappa-Β ligand (RANKL) signaling [100]. Similarly, USP13 and OTUD3 were downregulated in breast cancer tissues and were correlated with metastasis and poor survival. They both deubiquitinated a multifunctional tumor suppressor, phosphatase and tensin homolog (PTEN). Depletion of these DUBs can result in decreased PTEN expression and promote AKT signaling and tumorigenesis [101,102]. 

### 3.4. Apoptosis

**Figure 3:**
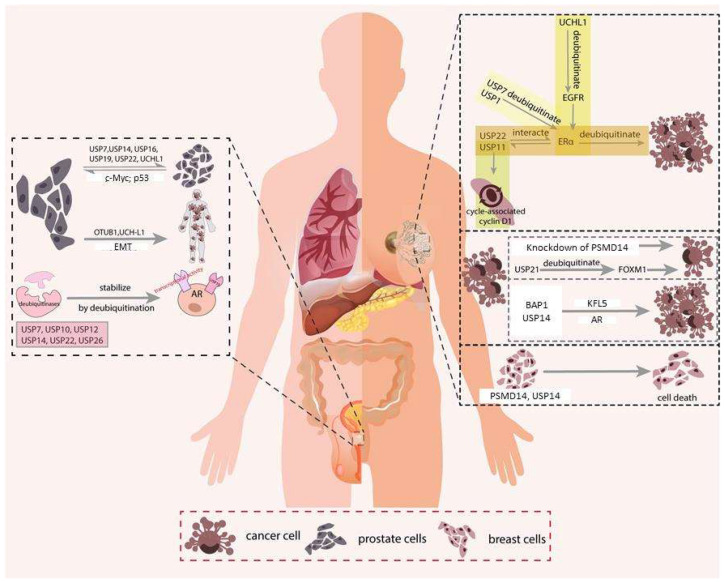
Roles of deubiquitinases (DUBs) in breast and prostatic cancer. The right panel describes the DUBs that related to the breast cancer. Breast cancer is linked to the ER signaling pathway, and the DUBs, such as UCHL1, USP1, USP7, USP11, and USP22 could regulate the ER signaling pathway and thus affect the cell cycle. Besides, DUBs such as BAP1, and USP14 act on KFL5 and AR respectively and play roles in proliferation. DUBs such as USP21 participate in cell death of the cancer cells. The left panel shows the prostatic cancer related DUBs. Prostatic cancer is linked to the AR signaling pathway. DUBs such as USP7, USP10, USP12, USP14, USP22, and USP26 stabilize the AR related proteins and affect various cellular events. In addition, DUBs such as USP7, USP14, USP16, USP19, USP22, and UCHL1 target on c-Myc and p53 that are related to the cell proliferation and cell death. Lastly, and DUBs such as OTUB1, and UCHL1 regulate the EMT and thus affect the metastasis. Detailed mechanisms can be referred to the main text.

USP14 plays an anti-apoptotic role in breast cancer via AR deubiquitination. In AR^+^ breast cancer cells, USP14 inhibition can induce poly (ADP-ribose) polymerase 1 (PARP) cleavage and suppress BCL2 protein expression [89]. Increased cleaved PARP levels have also been identified in the PSMD14 knockdown model, which leads to apoptosis [90]. A summary is shown in the right panel of Figure 3.

## 4. Prostate Cancer

Prostatic cancer is the second most common type of cancer among men, accounting for 14.1% of all cancers in men, and it is the second leading cause of cancer-related death in American men [103]. It develops via uncontrolled proliferation of semen-secreting prostate cells and is strongly linked to AR.

### 4.1. Androgen Receptor (AR)

The AR is a nuclear receptor that is activated by binding to various androgenic hormones, such as testosterone. Changes in AR expression or mutations are associated with prostate cancer [104]. DUBs such as USP7, USP12, USP14, USP22, and USP26 can interact with AR and thus increase its transcriptional activity via deubiquitination [105,106,107,108,109]. In addition, USP10 acts as an AR coactivator that can stimulate AR activity and androgen response of target promoters [110]. Co-factors related to DUBs have also been found to be related to cancer progression. For example, silencing of USP12 cofactors, Usp1-associated factor 1 (UAF1), or WD repeat domain 20 (WDR20), could influence the UAF1/WDR20/USP12 complex, thus inhibiting USP12 activity and AR-mediated transcription, leading to attenuated colony formation and promoting apoptosis [105].

### 4.2. Cell Proliferation

USP16 promote prostate cancer growth in vitro and in vivo by deubiquitinating the oncogene c-Myc. USP16 depletion in prostate cancer cells reduces cell proliferation, xenograft mass, and colony formation [111]. Other USP members, such as USP7, USP14, and USP22, have been found to promote cell proliferation, G0/G1 to S phase transition, and colony formation via AR in human prostate cancer cells [106,108,109]. In addition, depletion of USP19 reduces cell proliferation and causes cell arrest at the G_0_^/^G1 phase and p27^Kip1^ stabilization [112]. Furthermore, accumulation of p27^Kip1^ was found in weakly metastatic prostate cell lines in association with DUB UCHL1. UCHL1 suppresses cell proliferation through p53 stabilization and reduces Akt phosphorylation in prostate cancer [113]. 

### 4.3. Migration, Invasion & Metastasis

OTUB1 overexpress in prostate cancer tissues. In vitro studies have demonstrated that OTUB1 promotes prostate cancer invasion via RhoA activation. Prostate cancer cells transfected with OTUB1 shRNA exhibited delayed tumor growth, reduced tumor size, and metastasis in a mouse xenograft model [114]. In addition, UCHL1 is another key regulator of tumor metastasis [115], which is highly expressed in metastatic prostate cancer cell lines, but is not detected in weakly metastatic and benign prostate cancer cell lines. UCHL1 induces EMT, and thus enhances the migration and invasion processes in metastatic prostate cell lines [116]. On the other hand, there are other DUBs, such as USP9X and UCHL3, that reduce their expression in prostate cancers. In vitro prostate cancer cell line studies showed that the depletion of USP9X resulted in increased cell migration and invasion, which was achieved by the upregulated expression of ERK-mediated MMP9 and phosphorylated dynamin-related protein 1 (DRP1) [117]. Similarly, knockdown of UCHL3 promoted EMT in normal prostate cell lines and led to increased cell migration and invasion, whereas UCHL3 overexpression in prostate cancer cell lines reversed such processes [118]. 

### 4.4. Apoptosis

USP2a is highly expressed in prostate tumors and exerts anti-apoptotic effects. It deubiquitinates MDM2 and promotes p53 degradation [28]. USP2a also targets fatty acid synthase (FAS), which is overexpressed in prostate cancer and is associated with tumor progression and metastasis [119,120]. USP2a knockdown in prostate cancer cells resulted in upregulation of p53 and p21 and downregulation of FAS and MDM2 [121]. In contrast, USP7 act as a tumor suppressor by deubiquitinating and stabilizing p53, which induce apoptosis and inhibit cell growth. Deubiquitinases also contribute to resistance to genotoxic insults in prostate cancer. A study showed that USP22 could promote cellular survival upon irradiation by modulating ubiquitylation of the nucleotide excision repair protein xeroderma pigmentosum, complementation group C (XPC), which is responsible for DNA repair [122]. A summary is shown in the left panel of Figure 3.

## 5. Colorectal Cancer

Colorectal cancer (CRC) ranks second in terms of cancer-related mortality in developed countries. Based on the WHO data, it accounted for more than 0.9 M deaths in 2020 that marked it as the second most lethal cancers.

### 5.1. Wnt Signaling

Upregulation of the Wnt signaling pathway is a significant feature of CRC [123,124]. DUBs that regulate Wnt signaling can influence colon cancer progression. USP39 contributes to CRC growth and metastasis through the Wnt/β-catenin pathway. Studies have shown that USP39 knockout inhibits the migration and invasion of colon cancer cells. In addition, the expression of key proteins in the Wnt/β-catenin pathway is reduced, further affecting the growth and metastasis of CRC [125]. The USP7 inhibitor P5090 reduces the activity of Wnt signaling by enhancing ubiquitination and degradation of β-catenin, indicating its role in cancer progression [126]. USP6NL also regulates β-catenin accumulation. USP6NL knockdown results in G0/G1 cell cycle arrest and suppresses cell proliferation in CRC [127]. Another DUB, USP44, modulates Axin-1 protein by regulating β-catenin, c-Myc, and cyclin D1 in the Wnt/β-catenin pathway [128,129]. Furthermore, USP42 stabilizes zinc and ring finger 3 (ZNRF3)/ring finger protein 43 (RNF43) on the cell surface, which plays a role in paracrine Wnt signaling in colon cancer cells [130]. Lastly, USP22 was found to mediate CRC cytochemical resistance through the Wnt/β-catenin pathway [131].

### 5.2. Cell Proliferation

Overexpression of USP29 stimulates the proliferation of colorectal cancer cell lines by regulating the activity of the cancer marker nuclear protein Ki67 [30]. OTUD6A is upregulated in human colorectal cancer patients. It promotes regulation of mitochondrial morphology and tumor occurrence by stabilization of dynamin-related protein 1 (DRP1). OTUD6A deficiency could reduce mitochondrial fragmentation, thus inhibiting the proliferation of tumor cells and impairing the growth of heterogeneous transplant tumors [132]. USP43 is another DUB that is highly expressed in colorectal cancer tissue. It affects cell proliferation, colony formation, migration, invasion, and expression of EMT-related biomarkers via deubiquitination and stabilization of the zinc finger E-box-binding homeobox 1 (ZEB1) protein, which plays an important role in CRC function [133]. USP5 is involved in the growth of CRC cells via deubiquitinating its substrate Tu translation elongation factor (EF-Tu) [134].

Special AT-rich sequence-binding protein-1 (SATB1) abnormalities are associated with colon cancer [135]. The interaction between USP47 and special AT-rich sequence-binding protein-1 (SATB1) mediates the deubiquitination and stability of USP47. When USP47 was defective, the transcriptional activity of the SATB1 target gene was impaired, and the proliferation of colon cancer cells was inhibited in the mouse model [136]. In addition, USP22 promotes G1-S transformation by deubiquitinating and stabilizing the rate-limiting cyclin CCND1. Its overexpression promotes invasive growth of colon cancer cells [137]. Furthermore, USP1 plays a vital role in CRC cell survival, and its knockdown induces growth arrest at the G2/M phase of the cell cycle [138]. In addition, USP19 antagonizes ring finger protein 1 (RPF1)-mediated malic enzyme 1 (ME1) degradation through deubiquitination, which in turn promotes lipid metabolism associated with ERK2 activity and CRC development in human patients [139]. Lastly, the knockout of USP7 could inhibit the proliferation of CRC cells via the MDM2-p53 complex [126,140]. 

### 5.3. Migration, Invasion & Metastasis

More than 25% of colorectal cancer patients develop metastasis after diagnosis, which is the leading cause of death in CRC patients [141]. High OTUB1 expression in primary CRC tissue is associated with lymph node conditions and distant metastasis. OTUB1 promote the migration and invasion of CRC cells in vitro by altering EMT markers. It has been shown to induce liver metastasis of CRC cells in a mouse model [142]. In addition, PI3K/AKT/mTOR signaling activity is associated with invasion and poor tissue differentiation in CRC cells. Another DUB, UCHL3, regulates SRY-Box Transcription Factor 12 (SOX12) and participates in invasive migration by activating these pathways [143]. Moreover, NLR family pyrin domain containing 7 (NLRP7), a member of the nucleotide-binding oligomerization domain (NOD) -like receptor family, promotes proliferation and metastasis of tumor cells. DUB USP10 interact with it and catalyzes its deubiquitination in CRC cells [144]. In addition, it also interacts with the carcinogen Musashi-2 (MSI2) and regulates its expression [31]. Furthermore, USP11 acts as an oncogene and is overexpressed in CRC tissues. It also plays a role in the growth and metastasis of cancer cells. USP11 promotes CRC progression by stabilizing the protein phosphatase 1 catalytic subunit alpha (PPP1CA) via deubiquitination. USP 11 protected PPP1CA from proteasome-mediated degradation by activating the ERK/MAPK signaling pathway [145]. Furthermore, USP21 controls Fos-related antigen 1 (Fra-1) dependency on migration and intrusion activity by deubiquitinizing Fra-1 in colon cancer cells [146]. Moreover, depletion of another DUB, PSMD14, significantly decreased tumorigenesis of CRC cells in a xenograft model, and its expression was correlated with malignant progression and survival of CRC patients [147]. Lastly, the depletion of OTUD1 exacerbated colon cancer progression. It promotes transferrin receptor protein 1 (TFRC)-mediated iron transport by deubiquitinating and stabilizing iron-responsive element binding protein 2 (IREB2), resulting in increased reactive oxygen species (ROS) production and apoptosis [148].

### 5.4. Apoptosis 

Several DUBs have been found to regulate apoptotic proteins, such as B-cell lymphoma 2 (BCL2), BCL2 associated X protein (BAX), and myeloid-cell leukemia 1 (MCL1), in CRC. USP22 exerts tumor suppressor functions in CRC. The absence of USP22 resulted in increased activity of the apoptosis inhibitor mTOR and tumorigenic properties. This effect can be reversed by mTOR inhibitors [149]. In addition, USP1 knockdown reduced the expression of the anti-apoptotic proteins BCL2 and MCL1 [138]. Another DUB, USP44, promotes the apoptosis of CRC cells via Axin1 deubiquitination and the Wnt signaling pathway [128]. USP47 was found to bind to the transcription elongation factor A3 (TCEA3), which is regulated by BAX [150].

## 6. Pancreatic Cancer

Pancreatic cancer is one of the most aggressive solid tumors, and more than 85% of pancreatic tumor cases are classified as pancreatic ductal adenocarcinoma (PDAC) [151,152]. Due to its insidious onset and rapid progression, most patients are diagnosed at an advanced stage, making it one of the most lethal types of cancer with less than a 10% five-year survival rate [151,153]. 

### 6.1. Akt Signaling

USP49 has been identified as a novel modulator of the AKT pathway, which plays a key role in tumorigenesis and chemotherapy response in pancreatic cancer. USP49 deubiquitination stabilize the AKT-related scaffold protein FK506, binding protein of 51 kDa (FKBP51), which in turn enhances the ability of the PH domain and leucine-rich repeat protein phosphatase (PHLPP) to dephosphorylate AKT. In addition, it inhibits the proliferation of pancreatic cancer cells. Clinically, decreased USP49 expression in patients with pancreatic cancer is associated with decreased FKBP51 expression and increased phosphorylation of AKT [154]. Recently, a conserved F-box protein, Fbxo45, was shown to interact with USP49 in pancreatic cancer cells, resulting in increased cell viability and motility capacity [155].

### 6.2. Cell Proliferation

USP9X is downregulated in pancreatic PDAC cell lines and in over 50% of PDAC tumors. It serves as a tumor suppressor gene, and its expression is inversely correlated with metastasis and poor post-surgical survival [156]. USP9X knockdown in mouse PDAC cells suppressed anoikis, partially by disabling the Usp9x/Itch pathway. USP9X also cooperates with the proto-oncogene Kras^G12D^ to promote pancreatic tumorigenesis in vivo by rapidly developing advanced pancreatic intraepithelial neoplasia and microinvasive neoplasms [157]. In contrast, Liu et al. reported that USP9X is highly expressed in pancreatic cancers compared to adjacent non-cancerous tissues. Knockdown of USP9X in pancreatic cells reduced cell growth, migration, and invasion, downregulated EMT markers, and increased apoptosis in vitro and in vivo [158,159,160]. Another DUB, USP21, was upregulated in PDAC cells and was found to promote tumor growth in vivo. It deubiquitinated transcription factor 7 (TCSF7) and subsequently promoted cancer cell stemness through upregulation of the Wnt/β-catenin pathway [161]. USP5 deubiquitinates the tumor suppressor Wilms tumor 1 (WT1) and promotes cell proliferation [162]. Other DUBs have been found to regulate the cell cycle in pancreatic cancer. USP28 promotes cancer cell growth by promoting cell cycle progression and inhibiting apoptosis via FOXM1-mediated Wnt/β-catenin signaling [163], whereas USP16 regulates chromosomal condensation and G2/M progression by deubiquitinating histone H2A and polo-like kinase 1 [164,165]. In addition, USP22 was shown to induce cell cycle protein-dependent kinase inhibitor 1A (CDKN1A) in pancreatic cancer, and MDM2 inhibitors enhanced the anti-pancreatic cancer effect of USP22 overexpression [166]. 

### 6.3. Migration, Invasion & Metastasis

Several DUBs are highly expressed in pancreatic cancer. USP18 upregulates in pancreatic cancer tissues compared to adjacent non-tumor tissues. It deubiquitinate Notch 1, increasing Notch1-dependent c-Myc expression and promoting cancer progression by reducing cell cycle arrest and apoptosis [167]. In addition, elevated USP5 expression in PDAC cells is associated with tumor metastasis. It deubiquitinate WT1, which is also overexpressed in PDAC cells, enhance tumor formation in a mouse xenograft model, and induce cell migration in vitro [162]. Protein disulfide isomerase family A member 6 (PDIA6) interacts with DUB COPS5 and contributes to pancreatic cancer progression. Its overexpression promotes deubiquitination of β-catenin and programmed death-ligand 1 (PD-L1) and subsequently upregulates their expression in cancer cells [168]. Finally, UCHL3 deubiquitinate and stabilize the proliferation-associated transcription factor FOXM1 and promote the invasiveness of pancreatic cancer cells [169]. 

### 6.4. Apoptosis

USP17 and OTUD1 are involved in regulating nuclear factor erythroid 2–related factor 2 (NRF2) and yes-associated protein 1 (YAP) protein levels that inhibit apoptosis. The expression of NRF2 and YAP in pancreatic cancer cells was downregulated when USP17 or OTUD1 was mutated, suggesting that both could regulate apoptosis in cancer cells [170]. Inhibition of USP7 attenuates cell growth and induces cell death in PDAC. Such inhibitors enhance the antitumor effects of PARP inhibitors in a fructose-bisphosphatase 1 (FBP1)-dependent manner [171]. 

## 7. Lung Cancer

Non-small cell lung cancer (NSCLC) accounts for approximately 85% of all lung cancer cases. It is commonly diagnosed at a late stage and results in a low 5-year overall survival rate [172]. Based on the WHO 2020 data, it is the most lethal cancer in the world, and has caused approximately 1.8 M deaths. It is the second most common cancer that has 2.21 M new cases per year. 

### 7.1. Proliferation

OTUD3 is highly expressed in human lung cancer tissues, and its increased expression is linked to a poor survival rate. It deubiquitinates and stabilize the 78-kDa glucose-regulated protein (GRP78) and promotes cell proliferation [173]. USP17 is another DUB overexpressed in NSCLC tissues. It regulates cell cycle progression by deubiquitinating and stabilizing cyclin A, which is associated with NSCLC cell proliferation. Its depletion could cause the transition of the cell cycle from the G0/G1 to S phase [174]. Furthermore, USP17 also promotes lung cancer growth by increasing inflammation in cancer and stem cells via the macrophages/lung cancer cells/USP17 axis [175]. In addition, the poor survival rate of NSCLC is linked to the high expression of US26 [176]. USP28 overexpression induces cancer cell proliferation [177]. In addition, some DUBs regulate oncogenes in lung cancer. For example, USP18 localizes to and stabilizes the oncogene KRAS. KRAS is susceptible to degradation when mislocalized in the plasma membrane. By stabilizing KRAS, USP18 sustains KRAS signaling and promotes tumorigenesis by upregulating the growth regulator cyclin D1 [178]. In addition, USP21 promote NSCLC cell proliferation by deubiquitinating the oncogene YY1 [179]. USP9X was found to promote tumor formation and growth in a mouse xenograft model. It deubiquitinates prostaglandin-endoperoxide synthase (PTGES) in NSCLC. PTGES is highly expressed in NSCLC and acts as a key enzyme in prostaglandin E2 (PGE2) synthesis. Finally, USP10 influences the AKT signaling pathway and activates phosphatase and tensin homolog (PTEN) by blocking its K63-linked polyubiquitination, which in turn suppresses the growth of NSCLC [180,181]. 

### 7.2. Migration, Invasion & Metastasis

USP24 promotes cancer malignancy by inducing IL-6 by stabilizing p300 and beta-transducing repeats-containing proteins (β-TrCP) to boost histone-3 acetylation and NF-B while lowering DNA (cytosine-5)-methyltransferase 1 (DNMT1) in M2 macrophages and lung cancer cells [182]. Furthermore, it promotes cancer malignancy by stabilizing bromodomain-containing proteins (BRDs) [183]. USP10 deficiency can also enhance carcinogenesis. It deubiquitinates the versatile transcription factor KLF4 in aggressive malignancies. [184]. Moreover, OTUB2 stabilizes U2 small nuclear RNA auxiliary factor 2 (U2AF2) and induces carcinogenesis via the AKT/mTOR signaling pathway [185]. In contrast, USP4 expression was suppressed by the EMT marker SNAIL 1 in the later stages of lung cancer that affected migration [186]. Furthermore, USP4 stabilizes Twist1 and results in enhanced tumorsphere formation and lung cancer stemness [187]. USP9X affects EMT and stimulates migration in lung cancer cell lines. USP37 is a SNAIL-specific deubiquitinase that promotes cell migration and stabilizes c-Myc in lung cancer [35,188]. Another DUB, OTUD3, regulates the carboxyl terminus of Hsc70-interacting protein (CHIP), which influences lung cancer metastasis by suppressing the OTUD3-GRP78 signal axis [177]. Lastly, USP21 promotes NSCLC migration and invasiveness by deubiquitinating YY1 [179]. 

### 7.3. Apoptosis

**Figure 4:**
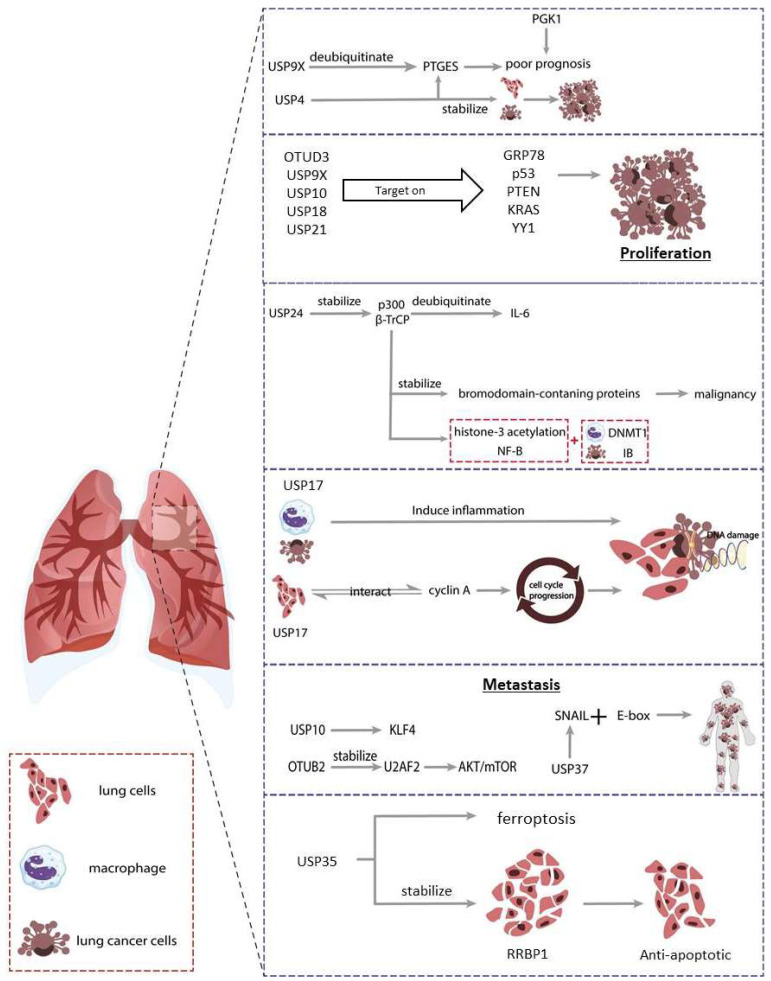
Roles of deubiquitinases (DUBs) in lung cancer. Various DUBs are found to be involved in lung cancer. DUBs control proliferation via various targets. For example, USP4, and USP9X target on PTGEs, while OTUD3, USP9X, USP10, USP18, and USP21 binds with GRP78, p53, PTEN, KRAS, and YY1 respectively. Besides, USP24 regulates inflammation and cell migration via stabilizing p300 and BRDs. Furthermore, USP17 induces inflammation and regulates cell cycle that links to DNA damage mechanism. Moreover, DUBs such as OTUB2, USP10, and USP37 regulate metastasis via U2AF2, KLF4, and SNAIL respectively. Lastly, USP35 stabilizes RRBP1 and controls the apoptosis. Detailed mechanisms can be referred back to the main text.

DNA repair is linked to apoptosis. Several DUBs, such as USP1 and USP35, are known to play roles in DNA repair [189]. USP35 stabilizes ribosome binding protein 1 (RRBP1) and reduces endoplasmic reticulum stress-induced apoptosis in NSCL [190]. It targets ferroportin and is related to ferroptosis [191]. Moreover, cell apoptosis is triggered by a reduction in USP28 [192]. USP7 controls the anti-tumor immune response by reprogramming tumor-associated macrophages [193]. USP10 deubiquitinates histone deacetylase 6 (HDAC6), which is highly expressed in NSCLC samples [194]. The low survival rate is linked to the c-Myc-USP10-p14ARF axis [195]. A summary is presented in Figure 4.

## 8. Conclusions

**Figure 5:**
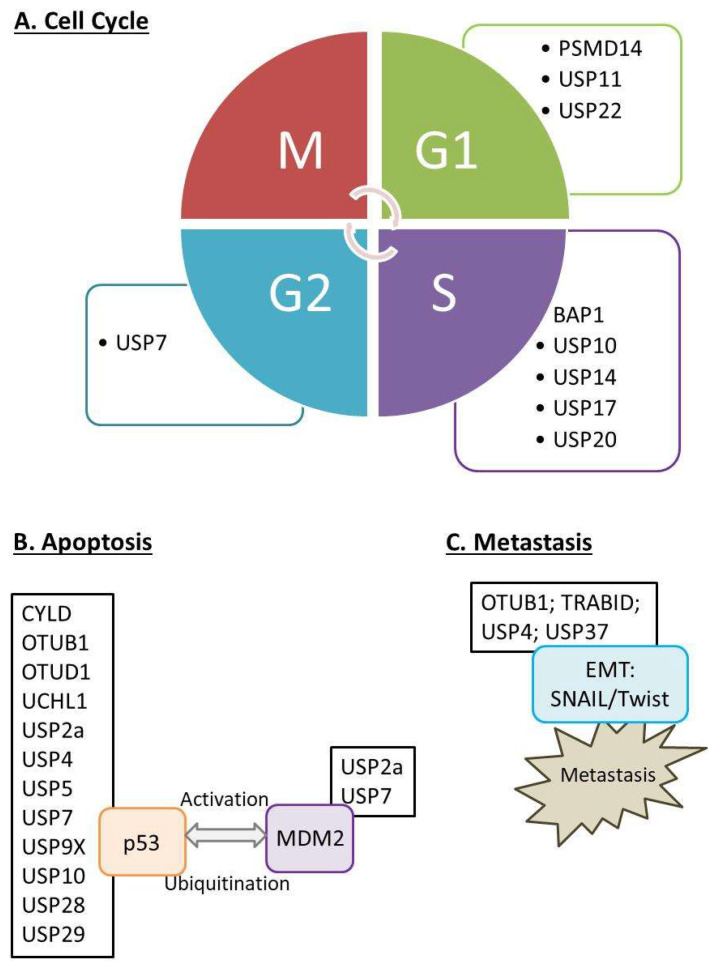
A brief summary of the roles of DUBs in this review. (**A**) DUBs affect cell cycle. Various cell cycle checkpoints are presented, and DUBs could regulate such events. PSMD1, USP11, and USP22 participate in G1 phase; while BAP1, USP10, USP14, USP17, and USP20 are involved in G1/S phase. USP7 is related to G2/M phase regulation. (**B**) DUBs regulate the apoptosis. Ubiquitination is occurred in both p53 and MDM2, and various DUBs could act on them. USP2a and USP7 are found to regulate both molecules. (**C**) EMT transcription factors such as SNAIL and TWIST are regulated by OTUB1, TRABID, USP4, and USP37. Detailed mechanisms can be referred back to the main text.

This review summarizes the current DUBs findings that correlate with different types of cancer. We believe that the current review can provide a quick guide for researchers to identify target DUBs in cancer. To conclude, we summarized the general roles of DUBs in terms of cell cycle (Figure 5A); apoptosis (Figure 5B); and metastasis (Figure 5C) and the selected targets of DUBs in this review for readers to have a quick reference (Table 1). As DUBs are involved in various biological processes, it is difficult to describe their detailed pathways in one article. Readers can refer to other reports for more specific and detailed mechanisms of particular cancers. DUBs are known to participate in cancer development; however, there are still many unknown mechanisms underlying their discovery. DUBs act on different targets, which allow them to influence various related signaling pathways and thus biological functions. In other words, it is difficult to specify the exact roles of DUBs in cancers. They can act as an initiator (its own mutation), promotor (act on target via deubiquitination) or enzymes (affect the activities). Nevertheless, by unfolding the underlying mechanisms and signaling pathways, it is generally accepted that targeting DUBs could be a potential therapy for treating cancer. The advancements in current omics and research tools could accelerate basic and clinical research, and we foresee that DUB–related small molecules might become a promising therapy for cancer treatment.

## Figures and Tables

**Table 1:** Targets of selected DUBs and their roles in cancers. P indicates the proliferation, M is metastasis, and A represents apoptosis.

Name	Targets	Functions
BAP1	KFL5	P
COPS5	β-catenin	M
CYLD	NEMO; TAK1; p53	P;M;A
OTUB1	SNAIL; p53	M
OTUB2	U2AF2	M
OTUD1	IREB2; p53	M;A
OTUD3	GRP78; PTEN	P;M
OTUD6A	DRP1	P
PSMD14	GRB2; TGFR; CAV1; CCND1	P;M;A
TRABID	Twist	M
UCHL1	EGFR; SMAD2; TGFR; p53	P;M;A
UCHL3	FOXM1	M
UCHL5	PRP19	M
USP1	ERα	P;A
USP10	KLF4; HDAC6; MSI2; NLPR7; PTEN; p53	P;M;A
USP11	Erα; CCND1; PPP1CA	P;M
USP12	AR	P
USP13	PTEN	M
USP14	AR	P;A
USP16	c-Myc; H2A	P
USP17	cyclin A; cMyc; p21	P
USP18	KRAS	P;M
USP19	p27; RPF1	P
USP20	SNAI2	M
USP21	MEK2; FOXM1; Fra-1; TCSF7; YY1	P;M
USP22	AR; CCND1; ERα; c-Myc	P;A
USP24	p300; BRDs	M
USP26	AR	P
USP28	FOXM1; p53; p21	P;A
USP29	p53	P
USP2a	MDM2; p53	A
USP35	RRBP1	A
USP37	SNAIL; c-Myc	M
USP4	PI3K; Twist1; p53; β-catenin	M
USP43	ZEB1	P
USP44	Axin1	A
USP47	SATB1; TCEA3	P;A
USP49	FKBP51	P
USP5	EF-Tu; WT1; p53	P;M
USP7	AR; ERα; MDM2; p53; β-catenin	P;A
USP9X	ASK1; PTGES; p53; β-catenin; survivin	P;M;A

## References

[1] Pickart C.M. (2001). Ubiquitin Enters the New Millennium. Mol. Cell.

[2] Hershko A., Ciechanover A. (1998). The ubiquitin system. Annu. Rev. Biochem..

[3] Aguilar R.C., Wendland B. (2003). Ubiquitin: Not just for proteasomes anymore. Curr. Opin. Cell Biol..

[4] Petroski M.D. (2008). The ubiquitin system, disease, and drug discovery. BMC Biochem..

[5] Nijman S.M.B., Luna-Vargas M.P.A., Velds A., Brummelkamp T.R., Dirac A.M.G., Sixma T.K., Bernards R. (2005). A genomic and functional inventory of deubiquitinating enzymes. Cell.

[6] Nijman S.M., Huang T.T., Dirac A.M., Brummelkamp T.R., Kerkhoven R.M., D’Andrea A.D., Bernards R. (2005). The Deubiquitinating Enzyme USP1 Regulates the Fanconi Anemia Pathway. Mol. Cell.

[7] Clague M.J., Barsukov I., Coulson J.M., Liu H., Rigden D.J., Urbe S. (2013). Deubiquitylases from Genes to Organism. Physiol. Rev..

[8] Rehman S.A.A., Kristariyanto Y.A., Choi S.-Y., Nkosi P.J., Weidlich S., Labib K., Hofmann K., Kulathu Y. (2016). MINDY-1 Is a Member of an Evolutionarily Conserved and Structurally Distinct New Family of Deubiquitinating Enzymes. Mol. Cell.

[9] Hermanns T., Pichlo C., Woiwode I., Klopffleisch K., Witting K.F., Ovaa H., Baumann U., Hofmann K. (2018). A family of unconventional deubiquitinases with modular chain specificity determinants. Nat. Commun..

[10] Liang J., Saad Y., Lei T., Wang J., Qi D., Yang Q., Kolattukudy P.E., Fu M. (2010). Mcp-induced protein 1 deubiquitinates traf proteins and negatively regulates jnk and nf-kappab signaling. J. Exp. Med..

[11] Komander D., Clague M.J., Urbe S. (2009). Breaking the chains: Structure and function of the deubiquitinases. Nat. Rev. Mol. Cell Biol..

[12] Eletr Z.M., Wilkinson K.D. (2013). Regulation of proteolysis by human deubiquitinating enzymes. Biochim. Biophys. Acta.

[13] Amerik A.Y., Hochstrasser M. (2004). Mechanism and function of deubiquitinating enzymes. Biochim. Biophys. Acta—Mol. Cell Res..

[14] Love K.R., Catic A., Schlieker C., Ploegh H.L. (2007). Mechanisms, biology and inhibitors of deubiquitinating enzymes. Nat. Chem. Biol..

[15] Song L., Rape M. (2008). Reverse the curse—The role of deubiquitination in cell cycle control. Curr. Opin. Cell Biol..

[16] Lai K.P., Chen J., Tse W.K.F. (2020). Role of Deubiquitinases in Human Cancers: Potential Targeted Therapy. Int. J. Mol. Sci..

[17] Hanahan D., Weinberg R.A. (2000). The hallmarks of cancer. Cell.

[18] Hanahan D., Weinberg R.A. (2011). Hallmarks of cancer: The next generation. Cell.

[19] Venuto S., Merla G. (2019). E3 Ubiquitin Ligase TRIM Proteins, Cell Cycle and Mitosis. Cells.

[20] Hussain S., Zhang Y., Galardy P.J. (2009). DUBs and cancer: The role of deubiquitinating enzymes as oncogenes, non-oncogenes and tumor suppressors. Cell Cycle.

[21] Vodermaier H.C. (2004). APC/C and SCF: Controlling Each Other and the Cell Cycle. Curr. Biol..

[22] Fernald K., Kurokawa M. (2013). Evading apoptosis in cancer. Trends Cell Biol..

[23] He M., Zhou Z., Wu G., Chen Q., Wan Y. (2017). Emerging role of DUBs in tumor metastasis and apoptosis: Therapeutic implication. Pharmacol. Ther..

[24] Bednash J.S., Mallampalli R.K. (2018). Targeting deubiquitinases in cancer. Methods Mol. Biol..

[25] Levine A.J. (1997). p53, the Cellular Gatekeeper for Growth and Division. Cell.

[26] Zhan T., Rindtorff N., Boutros M. (2017). Wnt signaling in cancer. Oncogene.

[27] Brooks C.L., Li M., Hu M., Shi Y., Gu W. (2007). The p53--mdm2--hausp complex is involved in p53 stabilization by hausp. Oncogene.

[28] Stevenson L.F., Sparks A., Allende-Vega N., Xirodimas D., Lane D., Saville M.K. (2007). The deubiquitinating enzyme USP2a regulates the p53 pathway by targeting Mdm2. EMBO J..

[29] Zhu L., Liu R., Zhang W., Qian S., Wang J.-H. (2015). MicroRNA-205 regulates ubiquitin specific peptidase 7 protein expression in hepatocellular carcinoma cells. Mol. Med. Rep..

[30] Chandrasekaran A., Suresh B., Sarodaya N., Ko N.-R., Oh S.-J., Kim K.-S., Ramakrishna S. (2021). Ubiquitin Specific Protease 29 Functions as an Oncogene Promoting Tumorigenesis in Colorectal Carcinoma. Cancers.

[31] Ouyang S.W., Liu T.T., Liu X.S., Zhu F.X., Zhu F.M., Liu X.N., Peng Z.H. (2019). Usp10 regulates musashi-2 stability via deubiquitination and promotes tumour proliferation in colon cancer. FEBS Lett..

[32] Potu H., Peterson L.F., Pal A., Verhaegen M., Cao J., Talpaz M., Donato N.J. (2014). Usp5 links suppression of p53 and FAS levels in melanoma to the BRAF pathway. Oncotarget.

[33] Boyer B., Vallés A.M., Edme N. (2000). Induction and regulation of epithelial-mesenchymal transitions. Biochem. Pharmacol..

[34] Nieto M.A., Huang R.Y., Jackson R.A., Thiery J.P. (2016). Emt: 2016. Cell.

[35] Cai J., Li M., Wang X., Li L., Li Q., Hou Z., Jia H., Liu S. (2019). USP37 Promotes Lung Cancer Cell Migration by Stabilizing Snail Protein via Deubiquitination. Front. Genet..

[36] Zhou H., Liu Y., Zhu R., Ding F., Cao X., Lin D., Liu Z. (2018). OTUB1 promotes esophageal squamous cell carcinoma metastasis through modulating Snail stability. Oncogene.

[37] Shen G., Lin Y., Yang X., Zhang J., Xu Z., Jia H. (2014). MicroRNA-26b inhibits epithelial-mesenchymal transition in hepatocellular carcinoma by targeting USP9X. BMC Cancer.

[38] Lv J., Zhang S., Wu H., Lu J., Lu Y., Wang F., Zhao W., Zhan P., Lu J., Fang Q. (2020). Deubiquitinase PSMD14 enhances hepatocellular carcinoma growth and metastasis by stabilizing GRB2. Cancer Lett..

[39] Bray F., Ferlay J., Soerjomataram I., Siegel R.L., Torre L.A., Jemal A. (2018). Global cancer statistics 2018: GLOBOCAN estimates of incidence and mortality worldwide for 36 cancers in 185 countries. CA Cancer J. Clin..

[40] Mahjoubin-Tehran M., De Vincentis A., Mikhailidis D.P., Atkin S.L., Mantzoros C.S., Jamialahmadi T., Sahebkar A. (2021). Non-alcoholic fatty liver disease and steatohepatitis: State of the art on effective therapeutics based on the gold standard method for diagnosis. Mol. Metab..

[41] Nowarski R., Gagliani N., Huber S., Flavell R.A. (2013). Innate Immune Cells in Inflammation and Cancer. Cancer Immunol. Res..

[42] Mantovani A., Allavena P., Sica A., Balkwill F. (2008). Cancer-related inflammation. Nature.

[43] Maeda H., Akaike T. (1998). Nitric oxide and oxygen radicals in infection, inflammation, and cancer. Biochem. Biokhimiia..

[44] Capece D., Fischietti M., Verzella D., Gaggiano A., Cicciarelli G., Tessitore A., Zazzeroni F., Alesse E. (2013). The inflammatory microenvironment in hepatocellular carcinoma: A pivotal role for tumor-associated macrophages. BioMed. Res. Int..

[45] Nakagawa H., Maeda S. (2012). Inflammation- and stress-related signaling pathways in hepatocarcinogenesis. World J. Gastroenterol..

[46] Pannem R.R., Dorn C., Ahlqvist K., Bosserhoff A.K., Hellerbrand C., Massoumi R. (2014). CYLD controls c-MYC expression through the JNK-dependent signaling pathway in hepatocellular carcinoma. Carcinogenesis.

[47] Hellerbrand C., Bumes E., Bataille F., Dietmaier W., Massoumi R., Bosserhoff A. (2007). Reduced expression of CYLD in human colon and hepatocellular carcinomas. Carcinogenesis.

[48] Urbanik T., Boger R.J., Longerich T., Becker K., Ehrenberg K.R., Hövelmeyer N., Hahn M., Schuchmann M., Jäger D., Waisman A. (2012). Liver specific deletion of cyldexon7/8 induces severe biliary damage, fibrosis and increases hepatocarcinogenesis in mice. J. Hepatol..

[49] Kovalenko A., Chable-Bessia C., Cantarella G., Israel A., Wallach D., Courtois G. (2003). The tumour suppressor cyld negatively regulates nf-kappa b signalling by deubiquitination. Nature.

[50] Trompouki E., Hatzivassiliou E., Tsichritzis T., Farmer H., Ashworth A., Mosialos G. (2003). Cyld is a deubiquitinating enzyme that negatively regulates nf-kappa b activation by tnfr family members. Nature.

[51] Xiao-Jing Z., Huang Z., Yan Z., Wang X., Zhao L.-P., Wang P.-X., Zhang X.-J., Alves-Bezerra M., Cai L., Zhang P. (2018). The deubiquitinating enzyme cylindromatosis mitigates nonalcoholic steatohepatitis. Nat. Med..

[52] Verboom L., Martens A., Priem D., Hoste E., Sze M., Vikkula H., Van Hove L., Voet S., Roels J., Maelfait J. (2020). OTULIN Prevents Liver Inflammation and Hepatocellular Carcinoma by Inhibiting FADD- and RIPK1 Kinase-Mediated Hepatocyte Apoptosis. Cell Rep..

[53] Damgaard R.B., Jolin H.E., Allison M.E.D., Davies S.E., Titheradge H.L., McKenzie A.N.J., Komander D. (2020). OTULIN protects the liver against cell death, inflammation, fibrosis, and cancer. Cell Death Differ..

[54] Li W., Cui K., Prochownik E.V., Li Y. (2018). The deubiquitinase USP21 stabilizes MEK2 to promote tumor growth. Cell Death Dis..

[55] Zhang Y., Jia J., Jin W., Cao J., Fu T., Ma D., Zhang Y. (2020). Lidocaine inhibits the proliferation and invasion of hepatocellular carcinoma by downregulating USP14 induced PI3K/Akt pathway. Pathol.—Res. Pract..

[56] Huang G., Li L.M., Zhou W.P. (2015). USP14 activation promotes tumor progression in hepatocellular carcinoma. Oncol. Rep..

[57] Yu J., Tao Q., Cheung K.F., Jin H., Poon F.F., Wang X., Li H., Cheng Y.Y., Röcken C., Ebert M.P.A. (2008). Epigenetic identification of ubiquitin carboxyl-terminal hydrolase L1 as a functional tumor suppressor and biomarker for hepatocellular carcinoma and other digestive tumors. Hepatology.

[58] Li M., Brooks C.L., Kon N., Gu W. (2004). A Dynamic Role of HAUSP in the p53-Mdm2 Pathway. Mol. Cell.

[59] Li M., Chen D., Shiloh A., Luo J., Nikolaev A.Y., Qin J., Gu W. (2002). Deubiquitination of p53 by HAUSP is an important pathway for p53 stabilization. Nature.

[60] Cummins J.M., Vogelstein B. (2004). HAUSP is Required for p53 Destabilization. Cell Cycle.

[61] Zhang J., Cao M., Dong J., Li C., Xu W., Zhan Y., Wang X., Yu M., Ge C., Ge Z. (2014). ABRO1 suppresses tumourigenesis and regulates the DNA damage response by stabilizing p53. Nat. Commun..

[62] Fang Y., Fu D., Tang W., Cai Y., Ma D., Wang H., Xue R., Liu T., Huang X., Dong L. (2013). Ubiquitin C-terminal Hydrolase 37, a novel predictor for hepatocellular carcinoma recurrence, promotes cell migration and invasion via interacting and deubiquitinating PRP19. Biochim. Biophys. Acta.

[63] Qiu C., Liu Y., Mei Y., Zou M., Zhao Z., Ye M., Wu X. (2018). Ubiquitin-specific protease 4 promotes metastasis of hepatocellular carcinoma by increasing TGF-β signaling-induced epithelial-mesenchymal transition. Aging.

[64] Chen H., Yang F., Li X., Gong Z., Wang L.-W. (2018). Long noncoding RNA LNC473 inhibits the ubiquitination of survivin via association with USP9X and enhances cell proliferation and invasion in hepatocellular carcinoma cells. Biochem. Biophys. Res. Commun..

[65] Giubellino A., Burke T.R., Bottaro D.P. (2008). Grb2 signaling in cell motility and cancer. Expert Opin. Ther. Targets.

[66] Wang B., Xu X., Yang Z., Zhang L., Liu Y., Ma A., Xu G., Tang M., Jing T., Wu L. (2019). POH1 contributes to hyperactivation of TGF-β signaling and facilitates hepatocellular carcinoma metastasis through deubiquitinating TGF-β receptors and caveolin-1. eBioMedicine.

[67] Zhu Y., Qu C., Hong X., Jia Y., Lin M., Luo Y., Lin F., Xie X., Xie X., Huang J. (2019). Trabid inhibits hepatocellular carcinoma growth and metastasis by cleaving RNF8-induced K63 ubiquitination of Twist1. Cell Death Differ..

[68] Urbanik T., Köhler B.C., Boger R.J., Wörns M.A., Heeger S., Otto G., Hövelmeyer N., Galle P.R., Schuchmann M., Waisman A. (2011). Down-regulation of cyld as a trigger for nf-κb activation and a mechanism of apoptotic resistance in hepatocellular carcinoma cells. Int. J. Oncol..

[69] Liu H., Chen W., Liang C., Chen B.W., Zhi X., Zhang S., Zheng X., Bai X., Liang T. (2015). WP1130 increases doxorubicin sensitivity in hepatocellular carcinoma cells through usp9x-dependent p53 degradation. Cancer Lett..

[70] Nagai H., Noguchi T., Homma K., Katagiri K., Takeda K., Matsuzawa A., Ichijo H. (2009). Ubiquitin-like Sequence in ASK1 Plays Critical Roles in the Recognition and Stabilization by USP9X and Oxidative Stress-Induced Cell Death. Mol. Cell.

[71] Zhang N., Liu L., Dou Y., Song D., Deng H. (2016). Glycogen synthase kinase-3β antagonizes ROS-induced hepatocellular carcinoma cell death through suppression of the apoptosis signal-regulating kinase 1. Med Oncol..

[72] Sung H., Ferlay J., Siegel R.L., Laversanne M., Soerjomataram I., Jemal A., Bray F. (2021). Global Cancer Statistics 2020: GLOBOCAN Estimates of Incidence and Mortality Worldwide for 36 Cancers in 185 Countries. CA Cancer J. Clin..

[73] Hanker A.B., Sudhan D.R., Arteaga C.L. (2020). Overcoming Endocrine Resistance in Breast Cancer. Cancer Cell.

[74] Lumachi F., Luisetto G., Basso S.M., Basso U., Brunello A., Camozzi V. (2011). Endocrine therapy of breast cancer. Curr. Med. Chem..

[75] Liu Y., Ma H., Yao J. (2020). ERα, A Key Target for Cancer Therapy: A Review. OncoTargets Ther..

[76] Wang S., Zhong X., Wang C., Luo H., Lin L., Sun H., Sun G., Zeng K., Zou R., Liu W. (2020). USP22 positively modulates ERα action via its deubiquitinase activity in breast cancer. Cell Death Differ..

[77] Niu Z., Li X., Feng S., Huang Q., Zhuang T., Yan C., Qian H., Ding Y., Zhu J., Xu W. (2020). The deubiquitinating enzyme USP1 modulates ERα and modulates breast cancer progression. J. Cancer.

[78] Xia X., Liao Y., Huang C., Liu Y., He J., Shao Z., Jiang L., Dou Q.P., Liu J., Huang H. (2019). Deubiquitination and stabilization of estrogen receptor α by ubiquitin-specific protease 7 promotes breast tumorigenesis. Cancer Lett..

[79] Dwane L., O’Connor A.E., Das S., Moran B., Mulrane L., Pinto-Fernandez A., Ward E., Blümel A.M., Cavanagh B.L., Mooney B. (2020). A Functional Genomic Screen Identifies the Deubiquitinase USP11 as a Novel Transcriptional Regulator of ERα in Breast Cancer. Cancer Res..

[80] Chen X.-S., Wang K.-S., Guo W., Li L.-Y., Yu P., Sun X.-Y., Wang H.-Y., Guan Y.-D., Tao Y.-G., Ding B.-N. (2020). UCH-L1-mediated Down-regulation of Estrogen Receptor α Contributes to Insensitivity to Endocrine Therapy for Breast Cancer. Theranostics.

[81] Ben-Porath I., Thomson M.W., Carey V.J., Ge R., Bell G.W., Regev A., Weinberg R.A. (2008). An embryonic stem cell-like gene expression signature in poorly differentiated aggressive human tumors. Nat. Genet..

[82] Tong D., Czerwenka K., Heinze G., Ryffel M., Schuster E., Witt A., Leodolter S., Zeillinger R. (2006). Expression of *KLF5* is a Prognostic Factor for Disease-Free Survival and Overall Survival in Patients with Breast Cancer. Clin. Cancer Res..

[83] Takagi K., Miki Y., Onodera Y., Nakamura Y., Ishida T., Watanabe M., Inoue S., Sasano H., Suzuki T. (2012). Krüppel-like factor 5 in human breast carcinoma: A potent prognostic factor induced by androgens. Endocr.-Relat. Cancer.

[84] Qin J., Zhou Z., Chen W., Wang C., Zhang H., Ge G., Shao M., You D., Fan Z., Xia H. (2015). BAP1 promotes breast cancer cell proliferation and metastasis by deubiquitinating KLF5. Nat. Commun..

[85] Peters A.A., Buchanan G., Ricciardelli C., Bianco-Miotto T., Centenera M.M., Harris J.M., Jindal S., Segara D., Jia L., Moore N.L. (2009). Androgen Receptor Inhibits Estrogen Receptor-α Activity and Is Prognostic in Breast Cancer. Cancer Res..

[86] Cochrane D.R., Bernales S., Jacobsen B.M., Cittelly D.M., Howe E.N., D’Amato N.C., Spoelstra N.S., Edgerton S.M., Jean A., Guerrero J. (2014). Role of the androgen receptor in breast cancer and preclinical analysis of enzalutamide. Breast Cancer Res..

[87] Doane A.S., Danso M., Lal P., Donaton M., Zhang L., Hudis C., Gerald W.L. (2006). An estrogen receptor-negative breast cancer subset characterized by a hormonally regulated transcriptional program and response to androgen. Oncogene.

[88] Ni M., Chen Y., Lim E., Wimberly H., Bailey S.T., Imai Y., Rimm D.L., Liu X.S., Brown M. (2011). Targeting Androgen Receptor in Estrogen Receptor-Negative Breast Cancer. Cancer Cell.

[89] Liao Y., Xia X., Liu N., Cai J., Guo Z., Li Y., Jiang L., Dou Q.P., Tang D., Huang H. (2018). Growth arrest and apoptosis induction in androgen receptor-positive human breast cancer cells by inhibition of USP14-mediated androgen receptor deubiquitination. Oncogene.

[90] Luo G., Hu N., Xia X., Zhou J., Ye C. (2017). RPN11 deubiquitinase promotes proliferation and migration of breast cancer cells. Mol. Med. Rep..

[91] Laoukili J., Kooistra M.R.H., Brás A., Kauw J., Kerkhoven R.M., Morrison A., Clevers H., Medema R. (2005). FoxM1 is required for execution of the mitotic programme and chromosome stability. Nat. Cell Biol..

[92] Peng L., Hu Y., Chen D., Linghu R., Wang Y., Kou X., Yang J., Jiao S. (2016). Ubiquitin specific protease 21 upregulation in breast cancer promotes cell tumorigenic capability and is associated with the NOD-like receptor signaling pathway. Oncol. Lett..

[93] Arceci A., Bonacci T., Wang X., Stewart K., Damrauer J.S., Hoadley K., Emanuele M.J. (2019). FOXM1 Deubiquitination by USP21 Regulates Cell Cycle Progression and Paclitaxel Sensitivity in Basal-like Breast Cancer. Cell Rep..

[94] Liu S., González-Prieto R., Zhang M., Geurink P.P., Kooij R., Iyengar P.V., van Dinther M., Bos E., Zhang X., Le Dévédec S.E. (2020). Deubiquitinase activity profiling identifies uchl1 as a candidate oncoprotein that promotes tgfβ-induced breast cancer metastasis. Clin. Cancer Res. Off. J. Am. Assoc. Cancer Res..

[95] Li W., Shen M., Jiang Y.-Z., Zhang R., Zheng H., Wei Y., Shao Z.-M., Kang Y. (2020). Deubiquitinase USP20 promotes breast cancer metastasis by stabilizing SNAI2. Genes Dev..

[96] Wang B., Ma A., Zhang L., Jin W.-L., Qian Y., Xu G., Qiu B., Yang Z., Liu Y., Xia Q. (2015). POH1 deubiquitylates and stabilizes E2F1 to promote tumour formation. Nat. Commun..

[97] Song Y., Li S., Ray A., Das D.S., Qi J., Samur M.K., Tai Y.-T., Munshi N., Carrasco R.D., Chauhan D. (2017). Blockade of deubiquitylating enzyme Rpn11 triggers apoptosis in multiple myeloma cells and overcomes bortezomib resistance. Oncogene.

[98] Wang C.-H., Lu S.-X., Liu L.-L., Li Y., Yang X., He Y.-F., Chen S.-L., Cai S.-H., Wang H., Yun J.-P. (2018). POH1 Knockdown Induces Cancer Cell Apoptosis via p53 and Bim. Neoplasia.

[99] Yu W., Li J., Wang Q., Wang B., Zhang L., Liu Y., Tang M., Xu G., Yang Z., Wang X. (2019). Targeting POH1 inhibits prostate cancer cell growth and enhances the suppressive efficacy of androgen deprivation and docetaxel. Prostate.

[100] Hayashi M., Jono H., Shinriki S., Nakamura T., Guo J., Sueta A., Tomiguchi M., Fujiwara S., Yamamoto-Ibusuki M., Murakami K.-I. (2014). Clinical significance of CYLD downregulation in breast cancer. Breast Cancer Res. Treat..

[101] Yuan L., Lv Y., Li H., Gao H., Song S., Zhang Y., Xing G., Kong X., Wang L., Li Y. (2015). Deubiquitylase OTUD3 regulates PTEN stability and suppresses tumorigenesis. Nat. Cell Biol..

[102] Zhang J., Zhang P., Wei Y., Piao H.-L., Wang W., Maddika S., Wang M., Chen D., Sun Y., Hung M.-C. (2013). Deubiquitylation and stabilization of PTEN by USP13. Nat. Cell Biol..

[103] Siegel R.L., Miller K.D., Fuchs H.E., Jemal A. (2022). Cancer statistics, 2022. CA A Cancer J. Clin..

[104] Fujita K., Nonomura N. (2019). Role of Androgen Receptor in Prostate Cancer: A Review. World J. Men’s Health.

[105] McClurg U.L., Harle V.J., Nabbi A., Batalha-Pereira A., Walker S., Coffey K., Gaughan L., McCracken S.R., Robson C.N. (2015). Ubiquitin-specific protease 12 interacting partners Uaf-1 and WDR20 are potential therapeutic targets in prostate cancer. Oncotarget.

[106] Liao Y., Liu N., Hua X., Cai J., Xia X., Wang X., Huang H., Liu J. (2017). Proteasome-associated deubiquitinase ubiquitin-specific protease 14 regulates prostate cancer proliferation by deubiquitinating and stabilizing androgen receptor. Cell Death Dis..

[107] Dirac A.M., Bernards R. (2010). The Deubiquitinating Enzyme USP26 Is a Regulator of Androgen Receptor Signaling. Mol. Cancer Res..

[108] Chen S.-T., Okada M., Nakato R., Izumi K., Bando M., Shirahige K. (2015). The Deubiquitinating Enzyme USP7 Regulates Androgen Receptor Activity by Modulating Its Binding to Chromatin. J. Biol. Chem..

[109] Schrecengost R.S., Dean J.L., Goodwin J.F., Schiewer M.J., Urban M.W., Stanek T.J., Sussman R.T., Hicks J.L., Birbe R.C., Draganova-Tacheva R.A. (2014). USP22 Regulates Oncogenic Signaling Pathways to Drive Lethal Cancer Progression. Cancer Res..

[110] Faus H., Meyer H.-A., Huber M., Bahr I., Haendler B. (2005). The ubiquitin-specific protease USP10 modulates androgen receptor function. Mol. Cell. Endocrinol..

[111] Ge J., Yu W., Li J., Ma H., Wang P., Zhou Y., Wang Y., Zhang J., Shi G. (2021). USP16 regulates castration-resistant prostate cancer cell proliferation by deubiquitinating and stablizing c-Myc. J. Exp. Clin. Cancer Res..

[112] Lu Y., Bedard N., Chevalier S., Wing S.S. (2011). Identification of Distinctive Patterns of USP19-Mediated Growth Regulation in Normal and Malignant Cells. PLoS ONE.

[113] Ummanni R., Jost E., Braig M., Lohmann F., Mundt F., Barett C., Schlomm T., Sauter G., Senff T., Bokemeyer C. (2011). Ubiquitin carboxyl-terminal hydrolase 1 (UCHL1) is a potential tumour suppressor in prostate cancer and is frequently silenced by promoter methylation. Mol. Cancer.

[114] Iglesias-Gato D., Chuan Y.-C., Jiang N., Svensson C., Bao J., Paul I., Egevad L., Kessler B.M., Wikström P., Niu Y. (2015). OTUB1 de-ubiquitinating enzyme promotes prostate cancer cell invasion in vitro and tumorigenesis in vivo. Mol. Cancer.

[115] Kim H.J., Kim Y.M., Lim S., Nam Y.K., Jeong J., Lee K.-J. (2009). Ubiquitin C-terminal hydrolase-L1 is a key regulator of tumor cell invasion and metastasis. Oncogene.

[116] Jang M.J., Baek S.H., Kim J.H. (2011). UCH-L1 promotes cancer metastasis in prostate cancer cells through EMT induction. Cancer Lett..

[117] Zhang J., Wang J., Luan T., Zuo Y., Chen J., Zhang H., Ye Z., Wang H., Hai B. (2019). Deubiquitinase USP9X regulates the invasion of prostate cancer cells by regulating the ERK pathway and mitochondrial dynamics. Oncol. Rep..

[118] Song H.M., Lee J.E., Kim J.H. (2014). Ubiquitin C-terminal hydrolase-L3 regulates EMT process and cancer metastasis in prostate cell lines. Biochem. Biophys. Res. Commun..

[119] Rossi S., Graner E., Febbo P., Weinstein L., Bhattacharya N., Onody T., Bubley G., Balk S., Loda M. (2003). Fatty acid synthase expression defines distinct molecular signatures in prostate cancer. Mol. Cancer Res..

[120] Pflug B.R., Pecher S.M., Brink A.W., Nelson J.B., Foster B.A. (2003). Increased fatty acid synthase expression and activity during progression of prostate cancer in the TRAMP model. Prostate.

[121] Priolo C., Tang D., Brahamandan M., Benassi B., Sicinska E., Ogino S., Farsetti A., Porrello A., Finn S., Zimmermann J. (2006). The Isopeptidase USP2a Protects Human Prostate Cancer from Apoptosis. Cancer Res..

[122] McCann J.J., Vasilevskaya I.A., Neupane N.P., Shafi A.A., McNair C., Dylgjeri E., Mandigo A.C., Schiewer M.J., Schrecengost R.S., Gallagher P. (2020). USP22 Functions as an Oncogenic Driver in Prostate Cancer by Regulating Cell Proliferation and DNA Repair. Cancer Res..

[123] Yang Y., Weng W., Peng J., Hong L., Yang L., Toiyama Y., Gao R., Liu M., Yin M., Pan C. (2017). Fusobacterium nucleatum increases proliferation of colorectal cancer cells and tumor development in mice by activating toll-like receptor 4 signaling to nuclear factor-κb, and up-regulating expression of microrna-21. Gastroenterology.

[124] Abed J., Emgård J.E., Zamir G., Faroja M., Almogy G., Grenov A., Sol A., Naor R., Pikarsky E., Atlan K.A. (2016). Fap2 mediates fusobacterium nucleatum colorectal adenocarcinoma enrichment by binding to tumor-expressed gal-galnac. Cell Host Microbe.

[125] Yuan X., Sun X., Shi X., Wang H., Wu G., Jiang C., Yu D., Zhang W., Xue B., Ding Y. (2017). USP39 promotes colorectal cancer growth and metastasis through the Wnt/β-catenin pathway. Oncol. Rep..

[126] Li X., Kong L., Yang Q., Duan A., Ju X., Cai B., Chen L., An T., Li Y. (2020). Parthenolide inhibits ubiquitin-specific peptidase 7 (USP7), Wnt signaling, and colorectal cancer cell growth. J. Biol. Chem..

[127] Sun K., He S.B., Yao Y.Z., Qu J.G., Xie R., Ma Y.Q., Zong M.H., Chen J.X. (2019). Tre2 (usp6nl) promotes colorectal cancer cell proliferation via wnt/beta-catenin pathway. Cancer Cell Int..

[128] Huang T., Zhang Q., Ren W., Yan B., Yi L., Tang T., Lin H., Zhang Y. (2020). USP44 suppresses proliferation and enhances apoptosis in colorectal cancer cells by inactivating the Wnt/β-catenin pathway via Axin1 deubiquitination. Cell Biol. Int..

[129] Sloane M.A., Wong J.W., Perera D., Nunez A.C., Pimanda J.E., Hawkins N.J., Sieber O.M., Bourke M.J., Hesson L.B., Ward R.L. (2014). Epigenetic inactivation of the candidate tumor suppressor *USP44* is a frequent and early event in colorectal neoplasia. Epigenetics.

[130] Giebel N., de Jaime-Soguero A., García Del Arco A., Landry J.J.M., Tietje M., Villacorta L., Benes V., Fernández-Sáiz V., Acebrón S.P. (2021). Usp42 protects znrf3/rnf43 from r-spondin-dependent clearance and inhibits wnt signalling. EMBO Rep..

[131] Miao D., Wang Y., Jia Y., Tong J., Jiang S., Liu L. (2021). ZRANB1 enhances stem-cell-like features and accelerates tumor progression by regulating Sox9-mediated USP22/Wnt/β-catenin pathway in colorectal cancer. Cell. Signal..

[132] Shi L., Liu J., Peng Y., Zhang J., Dai X., Zhang S., Wang Y., Liu J., Long J. (2020). Deubiquitinase OTUD6A promotes proliferation of cancer cells via regulating Drp1 stability and mitochondrial fission. Mol. Oncol..

[133] Ye D.-X., Wang S.-S., Huang Y., Wang X.-J., Chi P. (2021). USP43 directly regulates ZEB1 protein, mediating proliferation and metastasis of colorectal cancer. J. Cancer.

[134] Xu X., Huang A., Cui X., Han K., Hou X., Wang Q., Cui L., Yang Y. (2019). Ubiquitin specific peptidase 5 regulates colorectal cancer cell growth by stabilizing Tu translation elongation factor. Theranostics.

[135] Zhao J., Tuo Y., Luo W., He S., Chen Y. (2018). Prognostic and Clinicopathological Significance of SATB1 in Colorectal Cancer: A Meta-Analysis. Front. Physiol..

[136] Yu L., Dong L., Wang Y., Liu L., Long H., Li H., Li J., Yang X., Liu Z., Duan G. (2019). Reversible regulation of SATB1 ubiquitination by USP47 and SMURF2 mediates colon cancer cell proliferation and tumor progression. Cancer Lett..

[137] Gennaro V.J., Stanek T.J., Peck A.R., Sun Y., Wang F., Qie S., Knudsen K.E., Rui H., Butt T., Diehl J.A. (2018). Control of CCND1 ubiquitylation by the catalytic SAGA subunit USP22 is essential for cell cycle progression through G1 in cancer cells. Proc. Natl. Acad. Sci. USA.

[138] Xu X., Li S., Cui X., Han K., Wang J., Hou X., Cui L., He S., Xiao J., Yang Y. (2019). Inhibition of Ubiquitin Specific Protease 1 Sensitizes Colorectal Cancer Cells to DNA-Damaging Chemotherapeutics. Front. Oncol..

[139] Zhu Y., Gu L., Lin X., Zhou X., Lu B., Liu C., Lei C., Zhou F., Zhao Q., Prochownik E.V. (2021). USP19 exacerbates lipogenesis and colorectal carcinogenesis by stabilizing ME1. Cell Rep..

[140] Sheng Y., Saridakis V., Sarkari F., Duan S., Wu T., Arrowsmith C.H., Frappier L. (2006). Molecular recognition of p53 and mdm2 by usp7/hausp. Nat. Struct. Mol. Biol..

[141] Dykstra M.A., Gimon T.I., Ronksley P.E., Buie W.D., MacLean A.R. (2021). Classic and Novel Histopathologic Risk Factors for Lymph Node Metastasis in T1 Colorectal Cancer: A Systematic Review and Meta-analysis. Dis. Colon Rectum.

[142] Zhou Y., Wu J., Fu X., Du W., Zhou L., Meng X., Yu H., Lin J., Ye W., Liu J. (2014). OTUB1 promotes metastasis and serves as a marker of poor prognosis in colorectal cancer. Mol. Cancer.

[143] Li J., Zheng Y., Li X., Dong X., Chen W., Guan Z., Zhang C. (2020). UCHL3 promotes proliferation of colorectal cancer cells by regulating SOX12 via AKT/mTOR signaling pathway. Am. J. Transl. Res..

[144] Li B., Qi Z.-P., He D.-L., Chen Z.-H., Liu J.-Y., Wong M.-W., Zhang J.-W., Xu E.-P., Shi Q., Cai S.-L. (2021). NLRP7 deubiquitination by USP10 promotes tumor progression and tumor-associated macrophage polarization in colorectal cancer. J. Exp. Clin. Cancer Res..

[145] Sun H., Ou B., Zhao S., Liu X., Song L., Liu X., Wang R., Peng Z. (2019). USP11 promotes growth and metastasis of colorectal cancer via PPP1CA-mediated activation of ERK/MAPK signaling pathway. eBioMedicine.

[146] Yun S.-I., Hong H.K., Yeo S.-Y., Kim S.-H., Cho Y.B., Kim K.K. (2020). Ubiquitin-Specific Protease 21 Promotes Colorectal Cancer Metastasis by Acting as a Fra-1 Deubiquitinase. Cancers.

[147] Seo D., Jung S.M., Park J.S., Lee J., Ha J., Kim M., Park S.H. (2019). The deubiquitinating enzyme PSMD14 facilitates tumor growth and chemoresistance through stabilizing the ALK2 receptor in the initiation of BMP6 signaling pathway. eBioMedicine.

[148] Song J., Liu T., Yin Y., Zhao W., Lin Z., Yin Y., Lu D., You F. (2021). The deubiquitinase OTUD1 enhances iron transport and potentiates host antitumor immunity. EMBO Rep..

[149] Kosinsky R.L., Zerche M., Saul D., Wang X., Wohn L., Wegwitz F., Begus-Nahrmann Y., Johnsen S.A. (2020). USP22 exerts tumor-suppressive functions in colorectal cancer by decreasing mTOR activity. Cell Death Differ..

[150] Hou X., Xia J., Feng Y., Cui L., Yang Y., Yang P., Xu X. (2021). USP47-Mediated Deubiquitination and Stabilization of TCEA3 Attenuates Pyroptosis and Apoptosis of Colorectal Cancer Cells Induced by Chemotherapeutic Doxorubicin. Front. Pharmacol..

[151] Hezel A.F., Kimmelman A.C., Stanger B.Z., Bardeesy N., DePinho R.A. (2006). Genetics and biology of pancreatic ductal adenocarcinoma. Genes Dev..

[152] Pietri E., Balsano R., Coriano M., Gelsomino F., Leonardi F., Bui S., Gnetti L., Valle R.D., Garajová I. (2021). The implication of liquid biopsies to predict chemoresistance in pancreatic cancer. Cancer Drug Resist..

[153] Pan Y., Tang H., Li Q., Chen G., Li D. (2022). Exosomes and their roles in the chemoresistance of pancreatic cancer. Cancer Med..

[154] Luo K., Li Y., Yin Y., Li L., Wu C., Chen Y., Nowsheen S., Hu Q., Zhang L., Lou Z. (2017). USP49 negatively regulates tumorigenesis and chemoresistance through FKBP51-AKT signaling. EMBO J..

[155] Wu L., Yu K., Chen K., Zhu X., Yang Z., Wang Q., Gao J., Wang Y., Cao T., Xu H. (2022). Fbxo45 facilitates pancreatic carcinoma progression by targeting USP49 for ubiquitination and degradation. Cell Death Dis..

[156] Liu W., Huo Y., Yang J., Fu X., Yang M., Tao L., Liu D., Zhang J., Hua R., Sun Y. (2018). Decreased expression of USP9X is associated with poor prognosis in Chinese pancreatic ductal adenocarcinoma patients. Oncol. Lett..

[157] Pérez-Mancera P.A., Initiative A.P.C.G., Rust A., Van Der Weyden L., Kristiansen G., Li A., Sarver A.L., Silverstein K.A.T., Grützmann R., Aust D. (2012). The deubiquitinase USP9X suppresses pancreatic ductal adenocarcinoma. Nature.

[158] Liu L., Yao D., Zhang P., Ding W., Zhang X., Zhang C., Gong S., Zhang Y., Wang J., Sun T. (2017). Deubiquitinase USP9X promotes cell migration, invasion and inhibits apoptosis of human pancreatic cancer. Oncol. Rep..

[159] Cox J.L., Wilder P.J., Wuebben E.L., Ouellette M.M., Hollingsworth M.A., Rizzino A. (2014). Context-dependent function of the deubiquitinating enzyme USP9X in pancreatic ductal adenocarcinoma. Cancer Biol. Ther..

[160] Pal A., Dziubinski M., Di Magliano M.P., Simeone D.M., Owens S., Thomas D., Peterson L., Potu H., Talpaz M., Donato N.J. (2018). Usp9x Promotes Survival in Human Pancreatic Cancer and Its Inhibition Suppresses Pancreatic Ductal Adenocarcinoma In Vivo Tumor Growth. Neoplasia.

[161] Hou P., Ma X., Zhang Q., Wu C.-J., Liao W., Li J., Wang H., Zhao J., Zhou X., Guan C. (2019). USP21 deubiquitinase promotes pancreas cancer cell stemness via Wnt pathway activation. Genes Dev..

[162] Li J., Li H., Zhu W., Zhou B., Ying J., Wu J., Zhang H., Sun H., Gao S. (2020). Deubiquitinase inhibitor degrasyn suppresses metastasis by targeting USP5-WT1-E-cadherin signalling pathway in pancreatic ductal adenocarcinoma. J. Cell. Mol. Med..

[163] Chen L., Xu Z., Li Q., Feng Q., Zheng C., Du Y., Yuan R., Peng X. (2021). USP28 facilitates pancreatic cancer progression through activation of Wnt/β-catenin pathway via stabilising FOXM1. Cell Death Dis..

[164] Joo H.-Y., Zhai L., Yang C., Nie S., Erdjument-Bromage H., Tempst P., Chang C., Wang H. (2007). Regulation of cell cycle progression and gene expression by H2A deubiquitination. Nature.

[165] Zhuo X., Guo X., Zhang X., Jing G., Wang Y., Chen Q., Jiang Q., Liu J., Zhang C. (2015). Usp16 regulates kinetochore localization of Plk1 to promote proper chromosome alignment in mitosis. J. Cell Biol..

[166] Ren D., Sun Y., Li D., Wu H., Jin X. (2022). USP22-mediated deubiquitination of PTEN inhibits pancreatic cancer progression by inducing p21 expression. Mol. Oncol..

[167] Feng L., Wang K., Tang P., Chen S., Liu T., Lei J., Yuan R., Hu Z., Li W., Yu X. (2020). Deubiquitinase USP18 promotes the progression of pancreatic cancer via enhancing the Notch1-c-Myc axis. Aging.

[168] Ma Y., Xia P., Wang Z., Xu J., Zhang L., Jiang Y. (2021). PDIA6 promotes pancreatic cancer progression and immune escape through CSN5-mediated deubiquitination of β-catenin and PD-L1. Neoplasia.

[169] Fan Y., Hu D., Li D., Ma C., Tang Y., Tao Q., Deng L., Tang D. (2021). Uchl3 promotes aerobic glycolysis of pancreatic cancer through upregulating ldha expression. Clin. Transl. Oncol..

[170] Grattarola M., Cucci M.A., Roetto A., Dianzani C., Barrera G., Pizzimenti S. (2021). Post-translational down-regulation of Nrf2 and YAP proteins, by targeting deubiquitinases, reduces growth and chemoresistance in pancreatic cancer cells. Free Radic. Biol. Med..

[171] Chen H., Zhu X., Sun R., Ma P., Zhang E., Wang Z., Fan Y., Zhou G., Mao R. (2020). Ubiquitin-specific protease 7 is a druggable target that is essential for pancreatic cancer growth and chemoresistance. Investig. New Drugs.

[172] Goldstraw P., Chansky K., Crowley J., Rami-Porta R., Asamura H., Eberhardt W.E., Nicholson A.G., Groome P., Mitchell A., Bolejack V. (2016). The IASLC Lung Cancer Staging Project: Proposals for Revision of the TNM Stage Groupings in the Forthcoming (Eighth) Edition of the TNM Classification for Lung Cancer. J. Thorac. Oncol..

[173] Du T., Li H., Fan Y., Yuan L., Guo X., Zhu Q., Yao Y., Li X., Liu C., Yu X. (2019). The deubiquitylase OTUD3 stabilizes GRP78 and promotes lung tumorigenesis. Nat. Commun..

[174] Hu B., Deng T., Ma H., Liu Y., Feng P., Wei D., Ling N., Li L., Qiu S., Zhang L. (2019). Deubiquitinase DUB3 Regulates Cell Cycle Progression via Stabilizing Cyclin A for Proliferation of Non-Small Cell Lung Cancer Cells. Cells.

[175] Lu C.H., Yeh D.W., Lai C.Y., Liu Y.L., Huang L.R., Lee A.Y., Jin S.C., Chuang T.H. (2018). Usp17 mediates macrophage-promoted inflammation and stemness in lung cancer cells by regulating traf2/traf3 complex formation. Oncogene.

[176] Wang X., Liu Z., Zhang L., Yang Z., Chen X., Luo J., Zhou Z., Mei X., Yu X., Shao Z. (2018). Targeting deubiquitinase USP28 for cancer therapy. Cell Death Dis..

[177] Zhang P., Li C., Li H., Yuan L., Dai H., Peng Z., Deng Z., Chang Z., Cui C.-P., Zhang L. (2020). Ubiquitin ligase CHIP regulates OTUD3 stability and suppresses tumour metastasis in lung cancer. Cell Death Differ..

[178] Mustachio L.M., Lu Y., Tafe L.J., Memoli V., Rodriguez-Canales J., Mino B., Villalobos P.A., Wistuba I., Katayama H., Hanash S.M. (2017). Deubiquitinase USP18 Loss Mislocalizes and Destabilizes KRAS in Lung Cancer. Mol. Cancer Res..

[179] Xu P., Xiao H., Yang Q., Hu R., Jiang L., Bi R., Jiang X., Wang L., Mei J., Ding F. (2020). The usp21/yy1/snhg16 axis contributes to tumor proliferation, migration, and invasion of non-small-cell lung cancer. Exp. Mol. Med..

[180] He Y., Jiang S., Mao C., Zheng H., Cao B., Zhang Z., Zhao J., Zeng Y., Mao X. (2021). The deubiquitinase USP10 restores PTEN activity and inhibits non-small cell lung cancer cell proliferation. J. Biol. Chem..

[181] Sun J., Li T., Zhao Y., Huang L., Sun H., Wu H., Jiang X. (2018). USP10 inhibits lung cancer cell growth and invasion by upregulating PTEN. Mol. Cell. Biochem..

[182] Wang Y.C., Wu Y.S., Hung C.Y., Wang S.A., Young M.J., Hsu T.I., Hung J.J. (2018). Usp24 induces il-6 in tumor-associated microenvironment by stabilizing p300 and β-trcp and promotes cancer malignancy. Nat. Commun..

[183] Wang S.-A., Young M.-J., Jeng W.-Y., Liu C.-Y., Hung J.-J. (2020). USP24 stabilizes bromodomain containing proteins to promote lung cancer malignancy. Sci. Rep..

[184] Wang X., Xia S., Li H., Wang X., Li C., Chao Y., Zhang L., Han C. (2020). The deubiquitinase USP10 regulates KLF4 stability and suppresses lung tumorigenesis. Cell Death Differ..

[185] Li J., Cheng D., Zhu M., Yu H., Pan Z., Liu L., Geng Q., Pan H., Yan M., Yao M. (2019). OTUB2 stabilizes U2AF2 to promote the Warburg effect and tumorigenesis via the AKT/mTOR signaling pathway in non-small cell lung cancer. Theranostics.

[186] Lai C.-Y., Yeh D.-W., Lu C.-H., Liu Y.-L., Chuang Y.-C., Ruan J.-W., Kao C.-Y., Huang L.-R., Chuang T.-H. (2020). Epigenetic Silencing of Ubiquitin Specific Protease 4 by Snail1 Contributes to Macrophage-Dependent Inflammation and Therapeutic Resistance in Lung Cancer. Cancers.

[187] Li F., Hu Q., He T., Xu J., Yi Y., Xie S., Ding L., Fu M., Guo R., Xiao Z.-X.J. (2020). The Deubiquitinase USP4 Stabilizes Twist1 Protein to Promote Lung Cancer Cell Stemness. Cancers.

[188] Pan J., Deng Q., Jiang C., Wang X., Niu T., Li H., Chen T., Jin J., Pan W., Cai X. (2015). USP37 directly deubiquitinates and stabilizes c-Myc in lung cancer. Oncogene.

[189] García-Santisteban I., Peters G.J., Giovannetti E., Rodríguez J.A. (2013). USP1 deubiquitinase: Cellular functions, regulatory mechanisms and emerging potential as target in cancer therapy. Mol. Cancer.

[190] Wang W., Wang M., Xiao Y., Wang Y., Ma L., Guo L., Wu X., Lin X., Zhang P. (2022). USP35 mitigates endoplasmic reticulum stress-induced apoptosis by stabilizing RRBP1 in non-small cell lung cancer. Mol. Oncol..

[191] Tang Z., Jiang W., Mao M., Zhao J., Chen J., Cheng N. (2021). Deubiquitinase USP35 modulates ferroptosis in lung cancer via targeting ferroportin. Clin. Transl. Med..

[192] Zhang L., Xu B., Qiang Y., Huang H., Wang C., Li D., Qian J. (2015). Overexpression of deubiquitinating enzyme USP 28 promoted non-small cell lung cancer growth. J. Cell. Mol. Med..

[193] Dai X., Lu L., Deng S., Meng J., Wan C., Huang J., Sun Y., Hu Y., Wu B., Wu G. (2020). USP7 targeting modulates anti-tumor immune response by reprogramming Tumor-associated Macrophages in Lung Cancer. Theranostics.

[194] Hu C., Zhang M., Moses N., Hu C.-L., Polin L., Chen W., Jang H., Heyza J., Malysa A., Caruso J.A. (2020). The USP10-HDAC6 axis confers cisplatin resistance in non-small cell lung cancer lacking wild-type p53. Cell Death Dis..

[195] Ko A., Han S.Y., Choi C.H., Cho H., Lee M.-S., Kim S.-Y., Song J.S., Hong K.-M., Lee H.-W., Hewitt S.M. (2018). Oncogene-induced senescence mediated by c-Myc requires USP10 dependent deubiquitination and stabilization of p14ARF. Cell Death Differ..

